# Gradient Copolymers: A Complex Comonomer Incorporation Reality behind the Perfect Ideal

**DOI:** 10.1021/acspolymersau.5c00147

**Published:** 2026-03-06

**Authors:** Robert Conka, Yoshi W. Marien, Kevin M. Van Geem, Paul H. M. Van Steenberge, Richard Hoogenboom, Dagmar R. D’hooge

**Affiliations:** † Laboratory for Chemical Technology (LCT), 26656 Ghent University, Technologiepark 125, Ghent 9052, Belgium; ‡ Supramolecular Chemistry Group, Centre of Macromolecular Chemistry (CMaC), Department of Organic and Macromolecular Chemistry, Ghent University, Krijgslaan 281-S4, Ghent 9000, Belgium; § Intelligence in Processes, Advanced Catalysts and Solvents (iPRACS), Faculty of Applied Engineering, University of Antwerp, Groenenborgerlaan 171, Antwerp 2020, Belgium; ∥ Centre for Textiles Science and Engineering (CTSE), Ghent University, Technologiepark 70a, Ghent 9052, Belgium

**Keywords:** gradient copolymers, structural deviation, compositional distribution, kinetic Monte Carlo simulations, poly(2-alkyl/aryl-2-oxazoline)s, cationic ring opening polymerizaton

## Abstract

Gradient copolymers, which feature a gradual transition in monomer composition along the polymer backbone, uniquely combine tunable material properties with inherent stochasticity at the molecular level, bridging the structure–property landscape between block and random copolymers. Their broad glass transition temperature range, self-assembly potential, and amphiphilic behavior, if they consist of hydrophilic and hydrophobic comonomer units, enable applications in damping materials, drug delivery, and cosmetics. Moreover, they are interesting potential substitutes for block copolymers based on their much simpler and cheaper production process. However, gradient copolymers are not as simple as often presumed because they emerge from less trivial monomer inclusion probability profiles that are determined by monomer reactivity ratios and/or feeding profiles. As a result, gradient copolymers exhibit significant compositional heterogeneity, even under idealized conditions (fast chain initiation; no side reactions; and no diffusional limitations). This perspective highlights the critical importance of compositional control and structural evaluation in gradient (tapered) copolymer synthesis, highlighting the relevance of calculating a set of structural deviation (SD) metrics using coupled matrix-based Monte Carlo (CMMC) simulations to assess structural quality. In parallel to experimental protocol development and design, SD metrics such as the average SD (⟨SD⟩), SD standard deviation (σ_SD_), SD skewness ( ${\mathrm{\mu ̃}}_{3,SD}$ ), and coefficient of variation (CV_SD_) can be used to assess whether improved synthesis protocols are worthwhile or not. For a given synthesis recipe, a simultaneous SD evaluation with respect to block, gradient, block–gradient, and block–gradient–block targets is recommended based on a framework calibrated on the individual chain level. This facilitates the identification of the application scope of both exploratory and systematic research on gradient copolymer synthesis approaches.

## Graphical abstract

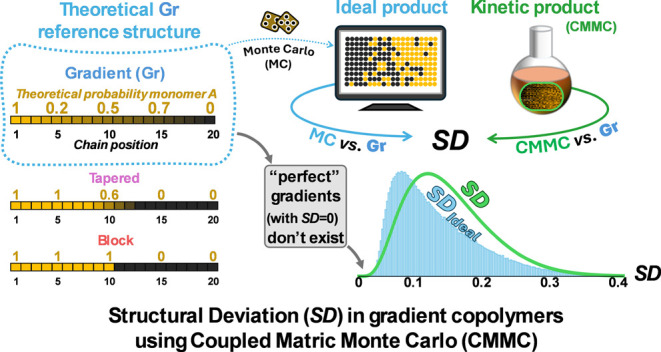

## 1 Introduction

Gradient copolymers are a unique class of materials that bridge the structural and functional properties of block and random copolymers.^1–5^ Characterized by a gradual transition in monomer composition along the polymer chains, they exhibit a broad glass transition temperature (*T*_g_),^6–9^ making them highly effective as damping materials.^10–13^ Their ability to self-assemble into supramolecular structures and micelles lends them great potential in applications, such as drug delivery and encapsulation,^5,14,15^ cosmetics, and personal care products.^16,17^ Gradient copolymers combine versatile compositional variations with tunable physical properties, explaining why both experimental and theoretical approaches are actively being explored to optimize their synthesis, and to deepen our understanding of their physical behavior.^3,18–23^ It should, however, be noted that direct characterization of monomer sequences at the single-chain level remains extremely challenging, and experimental studies typically rely on cumulative composition as a practical proxy.^24^

In general, these approaches deal with the targeting of well-defined block (Bl), tapered (Ta), or gradient (Gr) copolymer chains that differ in how their monomer composition evolves along the backbone (Figure 1). Because these structures are defined by a specific positional variation in monomer incorporation, it is essential to quantify how closely the synthesized chains follow their intended compositional profiles. Structural deviation (SD) provides a rigorous framework to assess this fidelity.

**Figure 1:**
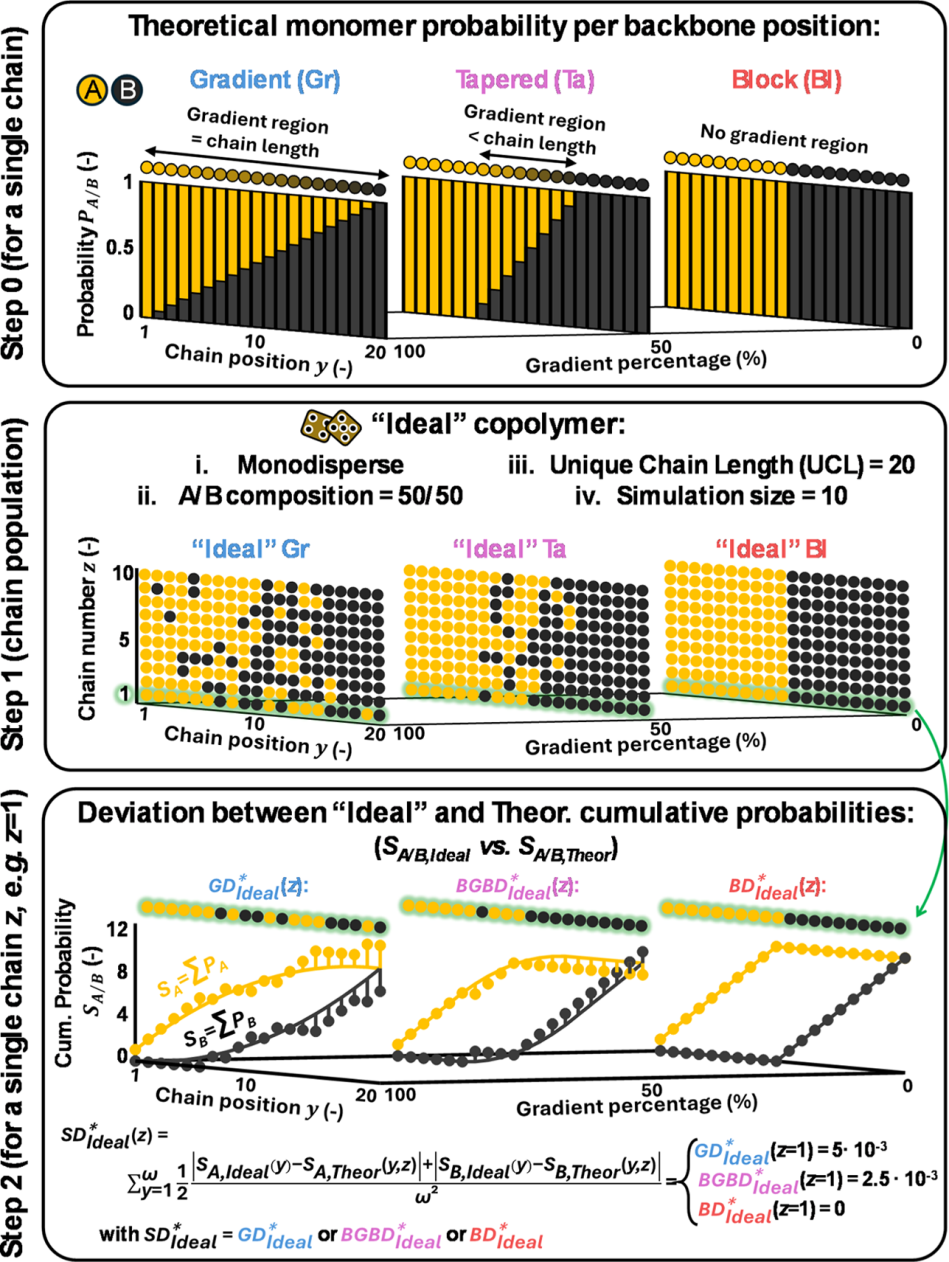
Schematic illustration of structural deviation (SD_Ideal_*) analysis in ideal samples for different copolymer architectures.^25–28^ Top: target compositional profiles for gradient (Gr, left), tapered (Ta, middle), and block (Bl, right) copolymers, where yellow and black bars represent monomers A and B, respectively. Middle: stochastic realization of ideal linear polymer samples (10 chains, unique chain length UCL = 20 for clarity), showing the resulting variability around the target composition. Bottom: chain-specific cumulative incorporations (*S*_A/B,Ideal_: spheres) compared to the theoretical cumulative profiles (*S*_A/B,Theor_: lines), used to compute absolute structural deviations for each architecture: gradient deviation (GD_Ideal_*), block–gradient–block deviation (BGBD_Ideal_*), and block deviation (BD_Ideal_*). For clarity, only a small sample of chains is shown; in practice, simulations typically involve 10^5^ chains with realistic lengths to minimize statistical noise. The full SD calculation procedure is detailed in Section S1 of the Supporting Information.

In this framework, the targeted composition is represented by position-dependent incorporation probabilities *P*_A/B,Theor_(*y*), which specify at each chain position the likelihood of inserting monomer A or B in a theoretical reference structure.^25–27^ These probabilities are constructed at the single-chain level (Figure 1, top) and satisfy two fundamental constraints: local normalization (*P*_A,Theor_(*y*) + *P*_B,Theor_(*y*) = 1) and global consistency with the overall A/B ratio, ensuring that the cumulative incorporations *S*_A/B,Theor_(*y*) converge to the desired composition.

The calculation of structural deviation (SD) begins by encoding the target compositional profile in the position-dependent probabilities *P*_A/B,Theor_(*y*), defining the intended gradient (Gr), tapered (Ta), or block (Bl) architecture (Step 0, Figure 1, top) in a theoretical reference molecule. An ideal monodisperse polymer sample is then generated stochastically (Step 1, Figure 1, middle), so that all chains share the same length (unique chain length, UCL; e.g. UCL = 20 in Figure 1, middle) and overall composition (e.g., A/B = 50/50 in Figure 1, middle), while individual sequences naturally fluctuate around the theoretical cumulative profiles *S*_A/B,Theor_ yielding *S*_A/B,Ideal_. In Figure 1, the UCL is deliberately kept small to ensure that the chain composition is clearly visible. Similarly, the simulation in the figure considers only 10 chains for clarity, whereas in practice, simulating on the order of 10^5^ chains is recommended to reduce stochastic noise.^25^ For each chain, the absolute structural deviation SD_Ideal_* quantifies the discrepancy between the ideal and theoretical cumulative incorporations (*S*_A/B,Ideal_ vs *S*_A/B,Theor_) along the entire chain, yielding a single-value measure of how faithfully the chain reproduces the target composition (Figure 1, bottom). Examples are shown for chain *z* = 1 corresponding to a gradient (Gr) target (left, brown), a tapered (Ta) target (middle, pink), and a block (Bl) target (right, blue).

An application that clearly highlights the relevance of designing copolymer compositions is the synthesis of amphiphilic copolymers that can elegantly self-assemble into nanostructures (e.g. micelles) that can enhance the solubility, stability, and controlled release of hydrophobic substances. This is interesting for instance for delivery systems,^15,29,30^ coating applications,^31,32^ and responsive materials.^33,34^ Notably, the micelle properties vary for block, gradient, and tapered copolymers, albeit in a rather nontraditional manner. Block copolymers form compact, stable micelles with slow exchange, whereas tapered copolymers produce more dynamic micelles with softened interfaces, and gradient copolymers yield diffuse, adaptive micelles with fast chain exchange and environmental responsiveness.^35–38^ However, gradient copolymers have also been reported to lead to smaller micelles than analogues block copolymer micelles due to loop formation of hydrophobic monomer rich regions in the hydrophilic part.^5,39,40^

The preparation of block, tapered, and gradient copolymers requires the use of a living or controlled chain-growth polymerization method, as uncontrolled methods like free radical polymerization would lead to composition drift between chains rather than a gradual change in monomer composition along a chain. Leading synthesis techniques to realize compositional control over polymer chains include reversible deactivation radical polymerization (RDRP) techniques, such as reversible addition–fragmentation chain transfer (RAFT) polymerization, atom transfer radical polymerization (ATRP), and nitroxide-mediated polymerization (NMP), as well as living polymerization techniques, such as living cationic ring opening polymerization (CROP) and living anionic polymerization (AP).^3,19,41–43^ In any case, a high initiation efficiency is crucial for the preparation of a defined structure, with ideally all chains starting to grow simultaneously to ensure uniform monomer exposure. Initiation delays cause chains to start polymerization at different times, resulting in intermolecular compositional variation.^44–46^

Gradient copolymers can be prepared by two main methods, namely, forced gradient copolymerization based on continuous or stepwise addition of one of the monomers during the polymerization process or spontaneous gradient copolymerization based on the inherent different reactivity of the comonomers (see Figure 2). The first method provides higher versatility and control over the gradient structure that is formed, whereby it is crucial to ensure good mixing during monomer addition. The spontaneous gradient copolymerization method is much simpler and more reproducible, providing a more reliable and robust preparation method for gradient copolymer synthesis.

**Figure 2:**
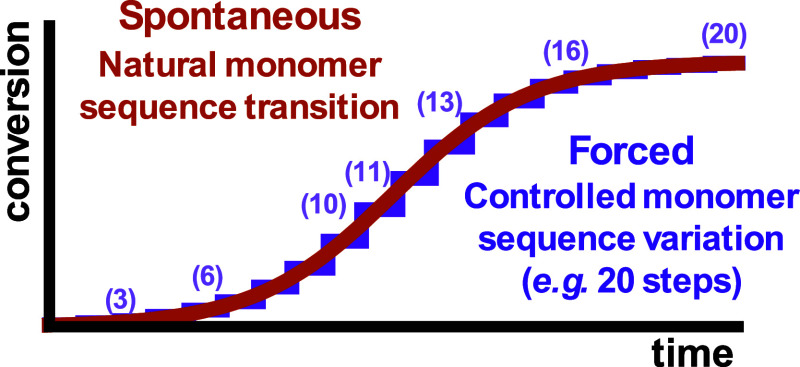
Conceptual time–conversion profiles for spontaneous (brown) vs forced (purple) gradient copolymerizations. Spontaneous gradients arise from inherent kinetic differences, while forced gradients are shaped by controlled monomer feed strategies.

For spontaneous gradient copolymerization, the (monomer) reactivity ratios *r* values, as influenced by the chemical structure of the monomers, significantly influence the gradient monomer distribution shape.^47^ If the monomer reactivity ratios differ strongly, compositional transitions occur naturally as one monomer is consumed faster than the other.^48,49^ This spontaneous gradient formation can be useful but may also result in (too) asymmetric profiles if not carefully controlled. Furthermore, the reactivity ratios govern the steepness and symmetry of the gradient that is formed, thereby limiting the possibility to vary the gradient structure, albeit synthesis conditions, such as temperature, solvent, and the (initial) (monomer) concentration(s), can affect chain propagation intensity and diffusivity.^50^ For instance, the temperature influences both reactivity and chain-end stability,^48,49^ while the solvent polarity impacts how readily monomers can reach the growing chains.^51,52^ These extra variables can be used to modulate the actual monomer inclusion probability profile that each chain experiences as a function of conversion, providing some control over the formed monomer gradients but also complicating uniform control over the formed polymers structures.

Model-based synthesis approaches have already been shown to offer simultaneous control over the gradient copolymer molecular structure and targeted thermal properties. For example, model-based design has been applied for semibatch ATRP of poly­(methyl methacrylate-grad-2-hydroxyethyl methacrylate) (poly­(MMA-*gr*-HEMA)) with control for composition profiles and *T*_g_ behavior.^53^ As thermal properties such as *T*_g_ are closely tied to chain composition, model-based monomer feeding strategies have become powerful tools for tailoring gradient copolymers, with related work focusing on including linear and nonlinear gradients, diblocks, and triblocks, through programmed semibatch reversible deactivation radical polymerization.^54^ Another recent study used RAFT polymerization design tools to reconstruct sequence ensembles via *k*MC simulations, with quantitative and visual metrics capturing local and global monomer patterns, providing a framework to design tailored, sequence-controlled polymers.^55^ Complementarily, a recent review highlighted that in silico modeling, including *k*MC simulations, provides powerful insights into gradient copolymer synthesis, allowing the prediction of monomer sequences, composition distributions, and thermal properties.^56^

As highlighted in the present scientific article, kinetic modeling, covering, e.g., systematic variations in feed rates, sets of reactivity ratios, and temperature profiles, allows the optimization of the synthesis conditions for the preparation of desired gradient shapes or target properties, such as *T*_g_, micelle size, or solubility. In particular, coupled matrix-based Monte Carlo (CMMC) is a kinetic Monte Carlo (*k*MC) simulation method that allows to explicitly model monomer sequence evolutions for individual chains with respect to synthesis time.^57–59^ In addition, structural deviation (SD) parameters can be calculated, which highlight if a given chain or ensemble of chains is close to a synthesis (e.g., gradient) target or not.^25,27,47,60^

This perspective first aims at creating broader awareness of the impossibility of making perfect gradient copolymer structures, as every gradient copolymer will inevitably consist of polymer chains that all differ in monomer gradient structures, which is often neglected and/or overlooked while it can be crucial for the properties. Second, we aim to make the experimental community more aware of CMMC modeling tools and SD parameters that are fully complementary with synthesis protocol development, polymer characterization, and experimental design. Even under perfectly controlled conditions, chain growth polymerization is inherently stochastic, introducing variability in intra- and interchain composition, whereby (i) the preparation of highly defined structures requires fine-tuned (likely low-efficiency) processes and (ii) scalable synthesis, typically leading to broader compositional distributions.

Characterizing compositional gradients in copolymers remains a significant analytical challenge. Unlike molar mass determination by size exclusion chromatography (SEC), no simple or standardized experimental method exists to directly assess the gradient quality. Current compositional analyses typically rely on practical proxies such as nuclear magnetic resonance (NMR), SEC, dynamic scanning calorimetry (DSC), or advanced chromatographic techniques, all of which provide ensemble-averaged structural information.^61–63^

The limitations of experimental resolution are, e.g., highlighted by studies using two-dimensional liquid chromatography under critical conditions (LCCC; 2D-LC) coupled with SEC (LCCC × SEC) on poly­(methyl acrylate) (polyMA)–polystyrene (polySt) block copolymers.^64^ These analyses provide information on both the chemical composition and molar mass for linear and star-shaped polyMA–polySt blocks. However, under critical conditions, the polyMA block becomes chromatographically “invisible” and the observed retention is dominated almost entirely by the polySt block. Changes in the molar mass or architecture of the polyMA block do not affect elution, demonstrating that even advanced 2D-LC can resolve block-level composition but cannot capture finer, monomer-scale details that define the sharpness of a gradient.

CMMC simulations offer a powerful approach to exploring gradient copolymer structures at the molecular level by tracking individual reaction events. These simulations are calibrated using experimentally accessible averaged properties, ensuring that the simulated chains reflect realistic polymer behavior. Once calibrated, CMMC simulations can provide quantitative insights into chain-to-chain variation, compositional distribution, and the quality of gradients or other structural features, information that is beyond the reach of experiments alone. Together, experimental characterization and calibrated CMMC simulations offer complementary perspectives: experiments anchor our understanding in measurable reality, while simulations extend this view to the molecular scale, providing a more mechanistic picture of how gradient structures form.

In what follows, the guidelines to in silico evaluate the quality of a synthesized copolymer with respect to a given target (e.g., gradient or tapered) will first be elaborated on, further opening doors to the synthesis of advanced materials with unique performance characteristics by a better understanding of monomer distributions and variations thereof, beyond a pure experimental approach. In a next step, it will be explained how chemical monomer design and synthesis conditions play a role in enhancing the compositional quality vs. a given synthesis target.

## 2 Guidelines for In Silico Gradient Quality Evaluation

### 2.1 Introducing Structural Deviation Metrics

The SD metric emerged in the 2010s to quantify how closely a set of monomer sequences of a polymer ensemble matches a targeted compositional profile. The gradient deviation (GD) metric, as introduced by Van Steenberge et al.^27^ in 2012, became the first member of the SD portfolio to assess gradient copolymer quality, to later on also include the block deviation (BD) metric to assess the (di) block copolymer structural quality.^26^ More recently the block–gradient deviation (BGD)^60^ as well as the block–gradient–block deviation (BGBD) metric^25^ have been introduced to assess the monomer incorporation quality for more hybrid structures. Initial emphasis has been on linear chains, while more recently, this has been expanded to chains with limited branching.^60^

Practically, an SD evaluation requires two inputs: (i) an ideal sample, constructed to match the targeted *P*_A/B,Theor_(*y*) and yielding *S*_A/B,Ideal_(*y*) for chains of a unique chain length and (ii) a real, kinetic sample obtained from CMMC simulations and represented by cumulative compositions *S*_A/B_(*y*). Consequently, the SD framework distinguishes two output metrics: (i) SD_Ideal_ obtained by comparing an ideal sample to its theoretical reference structure (*S*_A/B,Ideal_ vs *S*_A/B,Theor_) and (ii) SD obtained by comparing the real CMMC kinetic sample to the same theoretical reference structure (*S*_A/B_ vs *S*_A/B,Theor_). Normalization is achieved by dividing the raw non-normalized (SD_Ideal_*) value by the deviation obtained for a homopolymer (SD_HP_*) relative to the same target reference molecule. The different metrics related to the theoretical reference structure, ideal sample, and a real sample are summarized in Table S2 of the Supporting Information.

An average SD (⟨SD⟩) then results for the polymer ensemble sample (*z*_max_ chains), with values between 0 and 1. A low ⟨SD⟩ (close to 0) implies close alignment with the design target (minimum value of 0 for chains identical to the theoretical reference molecule), while a high ⟨SD⟩ reveals a substantial deviation in compositional fidelity (maximum value of 1 for a homopolymer). Hence, the ⟨SD⟩ value accounts for the polydisperse nature of polymers as well the impact of polymerization kinetics with main and side reactions, diffusional limitations, as well as various reactivity ratios. To also characterize the degree of molecular heterogeneity, it is recommended to calculate at least ⟨SD⟩ and a standard deviation σ_SD_. As highlighted below, it is even better to consider a set of SD metrics, i.e., ⟨SD⟩,σ_SD_, ${\mathrm{\mu ̃}}_{3,SD}$ , and CV_SD_, the latter two being the SD skewness and coefficient of variation.

### 2.2 Ideal or Targeted Polymer Compositions as First SD Calculation Input

Ideal polymer samples, which are generated via Monte Carlo (MC) sampling, rely solely on predefined *P*_A/B,Theor_ functions, producing monodisperse chains that are unaffected by kinetic, diffusive, or reactor operation constraints. An example of a 50% (symmetric) gradient with a perfectly controlled chain length of 100 is provided as Target T1 in Figure 3b. Here, all relevant SD metrics are designed specifically to evaluate gradient structures against T1, as illustrated in Figure 1 (left), and can therefore be interpreted directly as GD. An important observation for this ideal gradient copolymer is that it consists of a large ensemble of chains with different monomer distributions, already indicating that the perfect gradient copolymer does not exist. This is in contrast with an ideal block copolymer in which all block copolymer chains would have exactly the same composition when assuming perfect chain length control.

**Figure 3:**
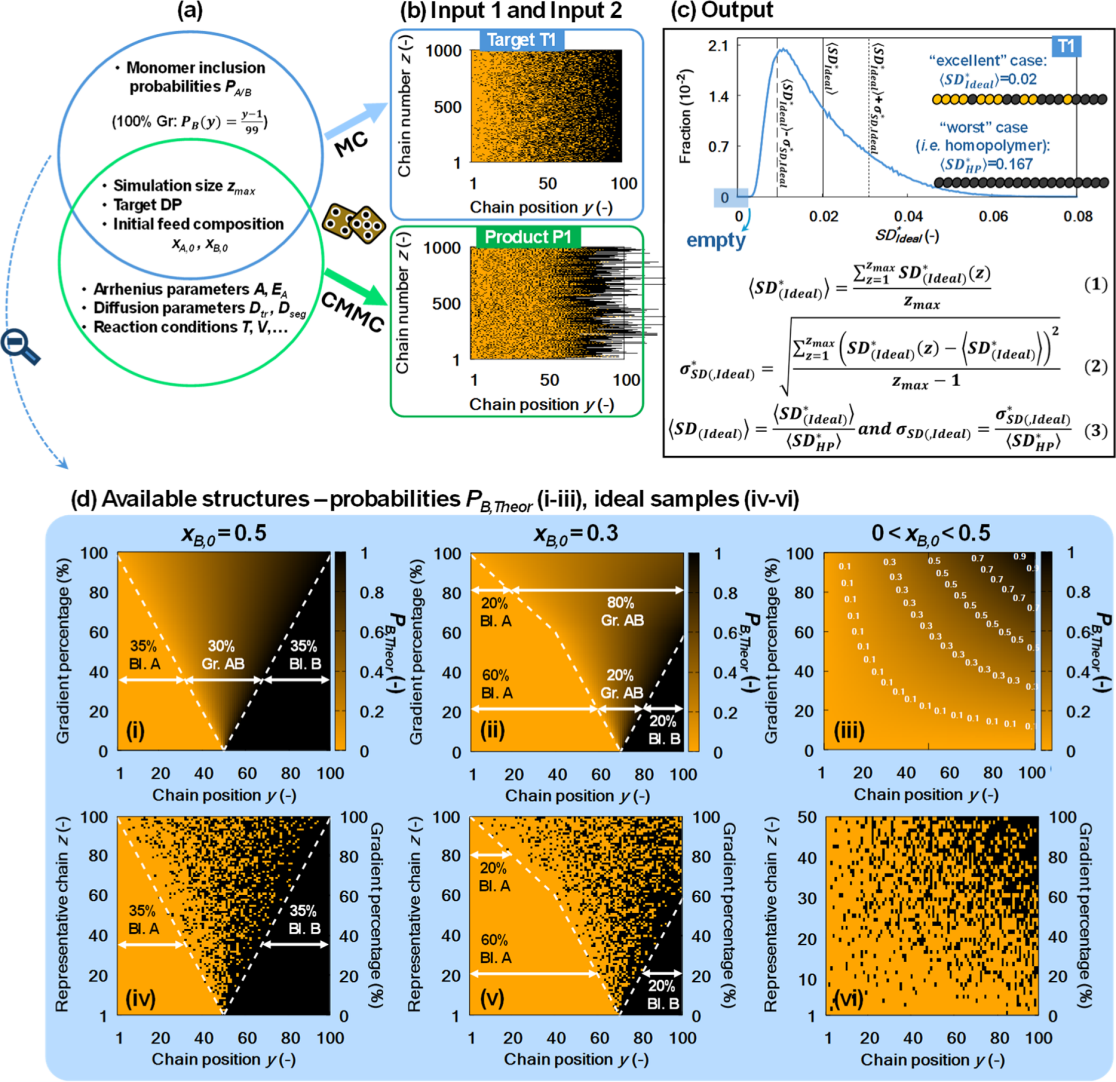
(a) Synthesis conditions/parameters to obtain structural deviations (SD) metrics, with, as example for the predefined monomer inclusion probabilities (*P*_A/B_), a 50% gradient target (blue T1; sample in subplot b top). An actual (in silico) synthesis result is given as P1 (green; sample in subplot b bottom). (c) Evaluation of the SD_Ideal_* distribution for T1, showing mean ⟨SD_Ideal_*⟩ (solid line) and ⟨SD_Ideal_*⟩ ± σ_SD,Ideal_* (dashed lines) via eq 2 and eq 3; “excellent” (high structural quality) and “worst” (homopolymer) cases marked and normalization via eq 4. (d) Extra examples on monomer inclusion probabilities for gradients, tapered structures, and blocks, with (i–iii) one chain for an ideal polymer sample in (iv–vi), for (i,iv) *x*_B,0_ = 0.5, (ii,v) *x*_B,0_ = 0.3, and (iii,vi) 0<*x*_B,0_ < 0.5. Extra SD_Ideal_* distributions in Figure 4. Adapted from European Polymer Journal, Vol. 185, Robert Conka, Yoshi W. Marien, Paul H.M. Van Steenberge, Richard Hoogenboom, Dagmar R. D’hooge, an equation driven quality classification of (a)­symmetric gradient, gradient-block, block-gradient-block and block copolymers, Article 111769, Copyright (2023), with permission from Elsevier.

A broader set of examples is included in Figure 3d, illustrating both the *P*_A/B,Theor_ functions (i–iii) and the corresponding ideal sample compositions (iv–vi) for symmetric and asymmetric targets across the gradient, tapered, and block copolymer spectrum. The actual calculation of ⟨SD_Ideal_⟩, starting from non-normalized SD_Ideal_* values for individual chains, is given in Figure 3c. Additional details on the SD calculations can be found in Sections S1 and S2 of the Supporting Information.

The compositional design specifications defined in panel i and panel ii of Figure 3d (target DP of 100) deliver the SD_Ideal_* distributions in Figure 4a,b, respectively. A more asymmetric case with *x*_B,0_ = 0.1 is included in Figure 4c (still target DP of 100) and the distributions for the design conditions shown in panel iii of Figure 3d are presented in Figure 4d (still target DP of 100, target Gradient percentage is 100%). Figure 4e in turn explores how variations in the target DP affect the SD_Ideal_* distributions for polymers targeting a 100% gradient. In all of the subplots in Figure 4, the ⟨SD_Ideal_*⟩ ± σ_SD,Ideal_* lines are also included, highlighting that a given compositional target is, even under ideal synthesis conditions, deviating from the 0 targeted value. Hence, it is impossible to make a perfect gradient or tapered structure and, in practice, one needs to be aware of and accept the inherent design deviations of gradient copolymers that will be further enlarged under actual synthesis conditions, where side reactions can also occur, leading to further deviation from the ideal target structure.

**Figure 4:**
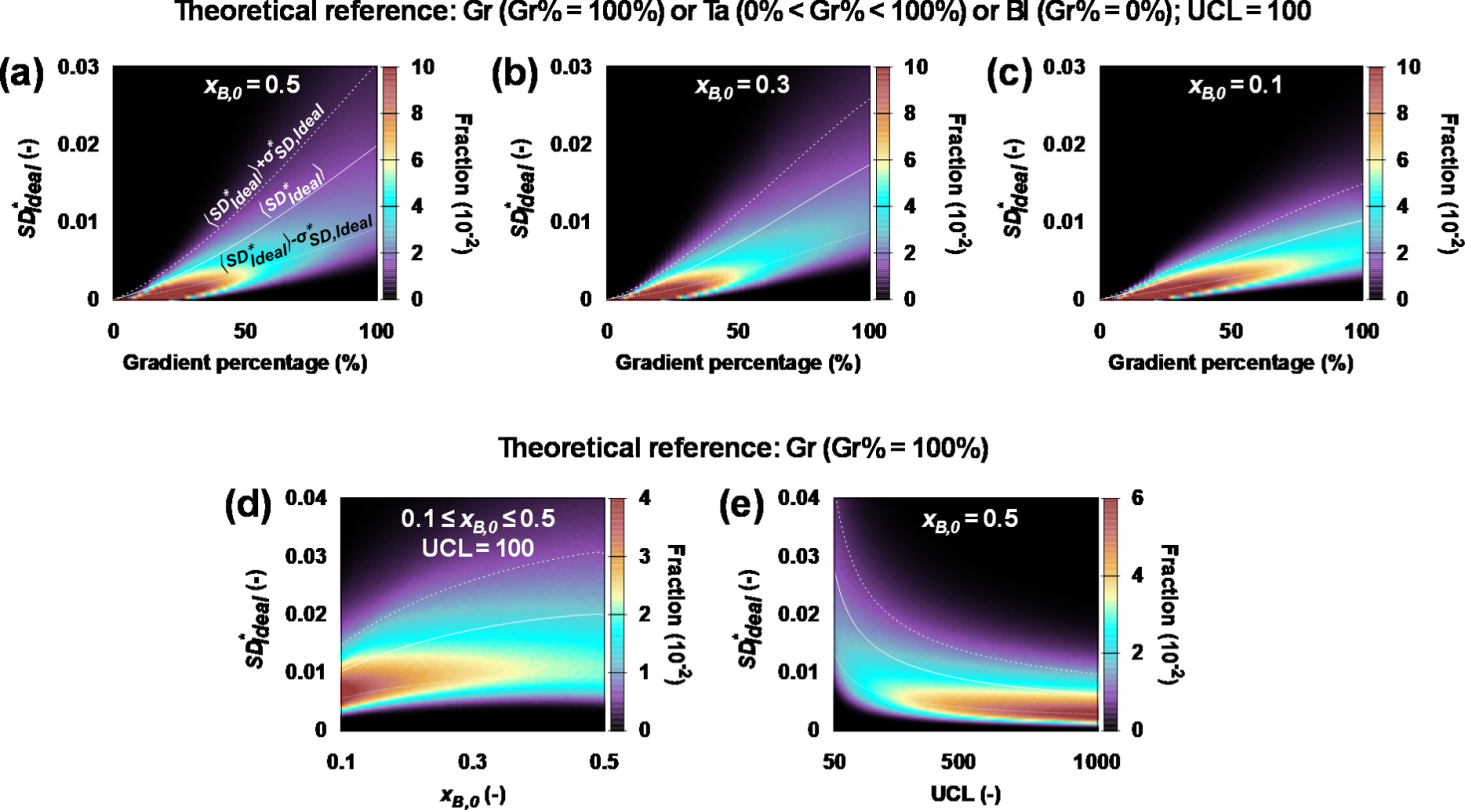
(a–c) Effect of gradient percentage (Gr­(adient) = 100%; 0%<Ta­(pered) < 100%; Bl­(ock) = 0%) on the absolute structural deviation (SD_Ideal_*) distribution, shown for 3 initial B fractions: (a) *x*_B,0_ = 0.5; (b) *x*_B,0_ = 0.3; and (c) *x*_B,0_ = 0.1. Decreasing the gradient percentage reduces both ⟨SD_Ideal_*⟩ and σ_SD,Ideal_* toward zero. At low *x*_B,0_, the gradient relevance starts to diminish, due to a minimal comonomer B content (*z*_max_ = 10^6^; target DP = 100). (d) Effect of the initial B fraction (0.1 ≤ *x*_*B*,0_ ≤ 0.5) on the absolute (gradient) structural deviation (SD_Ideal_* = GD_Ideal_*) distribution. Lower *x*_*B*,0_ values (i.e., lower symmetry) reduce ⟨SD_Ideal_*⟩ and σ_SD,Ideal_* but also diminish the relevance of gradient targeting, due to the low presence of comonomer B (*z*_max_ = 10^6^; 0.1 ≤*x*_*B*,0_ ≤ 0.5; gradient percentage = 100%; target DP = 100). (e) Effect of the target degree of polymerization (target DP) on the absolute (gradient) structural deviation (SD_Ideal_*) distribution. Increasing the target DP reduces ⟨SD_Ideal_*⟩ andσ _SD,Ideal_*, approaching a nonzero asymptote (*z*_max_ = 10^6^; *x*_*B*,0_ = *x*_*A*,0_ = 0.5; gradient percentage = 100%; 50 ≤ Target DP ≤ 1000). All subplots display ⟨SD_Ideal_*⟩ (solid line), ⟨SD_Ideal_*⟩ – σ_SD,Ideal_* (dotted line) and ⟨SD_Ideal_*⟩ + σ_SD,Ideal_* (dashed line). In subplots (d,e), all SD metrics coincide with the GD metric because the Gradient percentage is 100%. Reprinted in adapted from with permission from European Polymer Journal, 185, R. Conka, Y. W. Marien, P. H. M. Van Steenberge, R. Hoogenboom and D. R. D’hooge, an equation-driven quality classification of (a)­symmetric gradient, gradient–block, block–gradient–block and block copolymers, Article 111769, Copyright (2023), with permission from Elsevier.

Increasing the gradient percentage (Figure 4a–c) leads to higher ⟨SD_Ideal_⟩ values and broader SD_Ideal_ distributions, as perfect placement of monomer units in a more gradual A-to-B sequence becomes increasingly difficult. In contrast, tapered structures (with lower gradient percentages) are easier to realize under ideal conditions, resulting in narrower SD_Ideal_ distributions with lower ⟨SD_Ideal_*⟩ and σ_SD,Ideal_*, even approaching zero for the most blocky-like tapered polymers. For pure block copolymers (gradient percentage = 0%), both metrics reach zero, due to the aforementioned binary monomer inclusion probabilities (*P*_A/B,Theor_ and *P*_A/B,Ideal_ can only be 0 or 1), which leave no room for deviation.

From a comparison of Figure 4b,c with Figure 4a,d, it follows that gradient copolymer structures are more easily achieved in asymmetric copolymers. In the case one monomer is in clear excess, deviations from the target *P*_A/B_ profile have a smaller impact, as the dominant monomer unit largely determines the chain compositional structure. As a result, SD_Ideal_* distributions are narrower and more left-shifted (lower on the *y*-axis), with lower ⟨SD_Ideal_*⟩ and σ_SD,Ideal_* values, indicating improved structural alignment with the intended gradient. Nonetheless, these structures also have unavoidable intrinsic structural deviations in between different chains.

As shown in Figure 4e, increasing the target DP also improves the ideally possible gradient quality by lowering both ⟨SD_Ideal_*⟩ and σ_SD,Ideal_*. Longer chains provide more positions for gradual monomer placement, increasing the likelihood of approximating the ideal gradient sequence. Nonetheless, perfect gradient structures remain unattainable, as ⟨GD_Ideal_*⟩ and σ_GD,Ideal_* (i.e., ⟨SD_Ideal_*⟩ and σ_SD,Ideal_*for Gradient percentage not equal to zero) never fully reach zero, even at high target DP values.

### 2.3 Synthesized or Real Polymer Compositions as the Second SD Calculation Input

Examples of the compositions of real polymer samples are given in Figure 3 (product P1) as well as in Figure 5a as products P1–P4. These compositions have been generated using CMMC simulations according to ideal living polymerization conditions, i.e., without the occurrence of side reactions but considering a selection of monomer reactivity ratios (*r*_A_ and *r*_B_; see Section S3.2 of the Supporting Information), hence, different monomer combinations, and potentially a variation of polymerization conditions. The incorporation of more real-world deviations, such as a nonhomogenous temperature, diffusional limitations, or nonideal reactor behavior, leads to higher compositional distributions and increased structural deviations compared to the previous subsection. This becomes clear from inspecting the wider *x*-axis range in Figure 5b and the larger ⟨GD⟩ values in the table included in Figure 5c (values ≫ 0.01).

**Figure 5:**
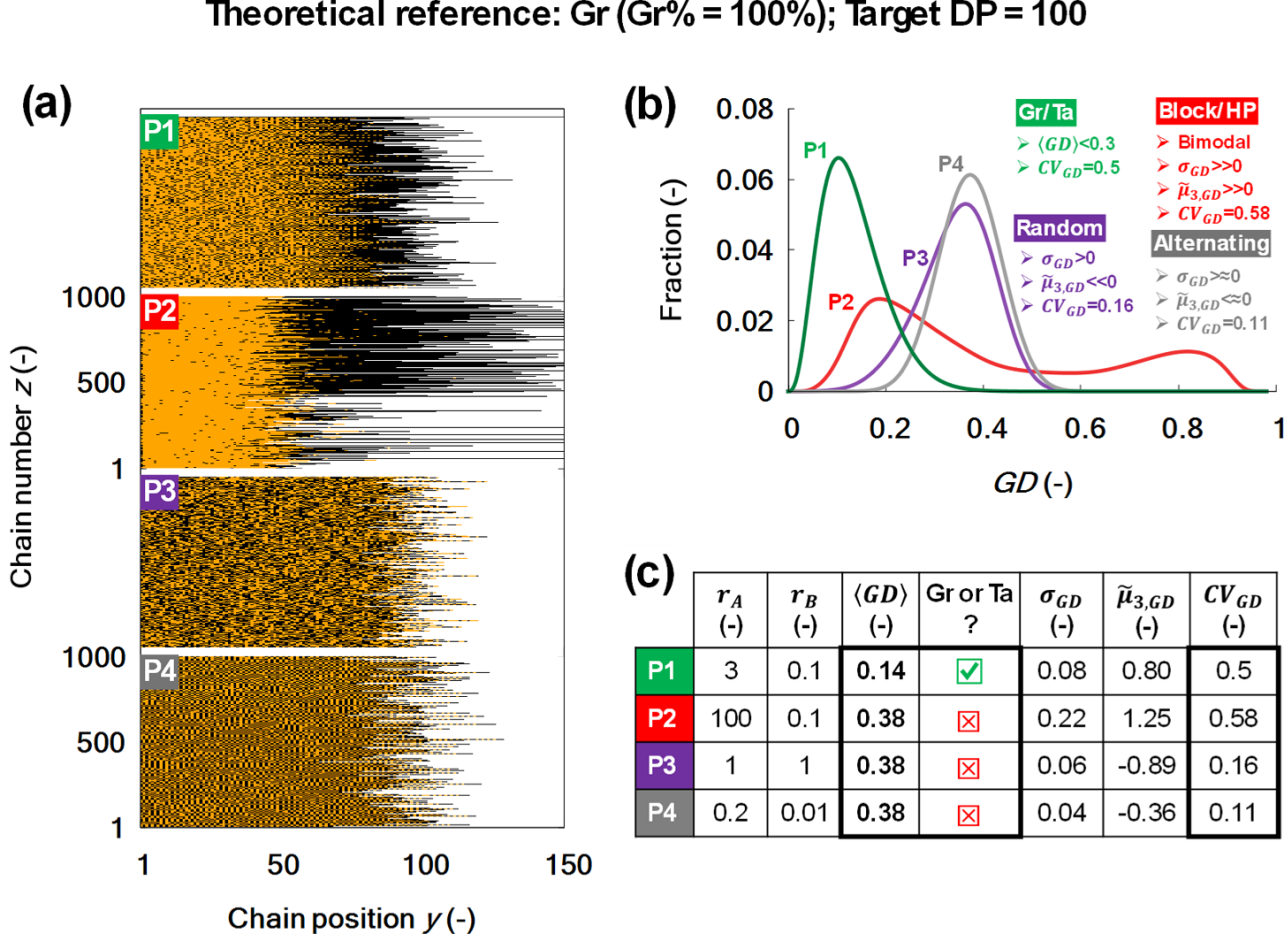
(a) Compositional representations of four in silico “real” polymer samples (P1–P4) with comonomers A (yellow) and B (black) (reaction scheme and model parameters in Section S3.1 of the Supporting Information). P1 exhibits a gradient structure, P2 has a block structure with a subpopulation of homopolymeric chains, P3 is a random copolymer, and P4 is an alternating copolymer. (b) Gradient deviation (GD) distributions for P1 (green), P2 (red), P3 (purple), and P4 (gray), with P2–P4 all yielding a ⟨GD⟩ of 0.38, suggesting similar compositions relative to the gradient target in case only ⟨GD⟩ is considered a metric. (c) The included table lists reactivity ratios *r*_A_ and *r*_B_, ⟨GD⟩, σ_**GD**_, CV_**GD**_, and ${\mathrm{\mu ̃}}_{3,GD}$ metrics for the polymer samples. The low ⟨GD⟩ in P1 confirms close alignment with the targeted gradient structure, while its high CV_GD_ value highlights the elevated structural variability associated with gradient copolymers. The P3 distribution is bimodal, with a strongly homopolymeric contribution (very positive ${\mathrm{\mu ̃}}_{3,GD}$ ), while P4 and P5 show left-skewed distributions, indicating random or alternating structures (negative ${\mathrm{\mu ̃}}_{3,GD}$ ). Hence, the metrics together enable an unbiased synthesis quality evaluation. Reprinted in adapted form with permission from R. Conka, Y. W. Marien, P. H. M. Van Steenberge, R. Hoogenboom and D. R. D’hooge, React. Chem. Eng*.*, 2023, 8, 2905–2923. Copyright (2023) Royal Society of Chemistry.

It follows that P1 is the closest to the targeted structure T1, as it displays the lowest ⟨GD⟩ value of 0.14 in Figure 5c. Indeed, P1 is deliberately generated in silico to mimic a gradient polymer, whereas P2–P4 are generated keeping different targets in mind. It should however be noted that P2–P4 display equally high ⟨GD⟩ values of 0.38, despite that a visual inspection of the copolymer samples in Figure 5a reveals very different monomer sequences. Figure 5a reveals that P2 features blocky chains with a homopolymeric subpopulation; P3 displays a random sequence, and P4 exhibits a near-alternating pattern. P2 is distinguishable from P3 and P4 due to its blocky nature, which retains a compositional transition, whereas the random (P3) and alternating (P4) sequences lack such transitions.

Hence, it is worthwhile, in a general context, to include additional SD parameters to be able to differentiate the synthesis success vs. a given (or series of) synthesis target(s) in an unbiased manner, as done in Figure 5. The differences between P2–P4 are specifically reflected in the structural deviation skewness ${\mathrm{\mu ̃}}_{3,SD}$ , here the gradient deviation skewness ${\mathrm{\mu ̃}}_{3,GD}$ . A right-skewed distribution for gradient copolymers delivers a positive skewness, highlighting a clear A-to-B (or B-to-A) transition and a right tail from homopolymeric­(-like) deviations. In contrast, random or alternating copolymers uniquely exhibit uniform or oscillatory comonomer incorporation, resulting in a left-skewed distribution, hence, negative skewness with most chains near the average deviation and fewer at higher deviations. Homopolymeric structures reinforce this left skew, due to minimal deviation from a uniform composition.^47^

The average gradient degree ⟨GD⟩ provides a reliable means of distinguishing gradient copolymers (low ⟨GD⟩) from nongradient structures, including homopolymeric, blocky, random, or alternating sequences (high ⟨GD⟩). ⟨GD⟩ alone thus does not convey the detailed monomer sequence arrangements within nongradient samples. In this context, the average primarily serves to identify gradients, whereas higher-order statistics are required to characterize nongradient architectures. Including mathematical information about the shape and broadness of the GD distribution offers estimative insights into chain organization.^47^

In nearly alternating or random-like structures with very low *r*_A_ and *r*_B_, chains exhibit minimal chain-wide A-to-B shifts due to continuous local A/B alternatives. Local deviations from (ideal) alternation produce short stretches enriched in either A or B that are otherwise absent in ideal alternation. These toggles introduce gradient-like features absent in purely alternating or highly random arrangements, such that chains closest to an ideal gradient occur with probabilities similar to slightly less optimal chains, because an increase in the number of toggles gives more gradient-like features.

In copolymerization with strongly mismatched rate coefficients (very large *r*_A_, very small *r*_B_, or vice versa), it struggles to incorporate both monomers at comparable rates. This produces a major subpopulation resembling a homopolymer of the faster-reacting monomer (reaching the target DP with little deviation), accompanied by a minor subpopulation containing long segments of the slower-reacting monomer formed only after the faster monomer is depleted. Effectively, the system splits into two homopolymer-like subpopulations, and in such cases, even sparse A/B toggles reduce the GD values of chains that would otherwise appear purely homopolymeric.

In both cases (alternating/random and homopolymeric), this behavior corresponds to negatively skewed distributions ( ${\mathrm{\mu ̃}}_{3,GD}$ <0), in which the tail extends toward lower GD values, reflecting that a fraction of chains spans a wider range of GD below the average than above it.

CV_GD_, defined as the ratio of σ_GD_ to ⟨GD⟩, helps to distinguish these cases because alternating copolymers arise from similarly low reactivity ratios; this similarity keeps the structural variation small, giving a low CV_GD_. Homopolymer-like systems, however, show the opposite behavior: they consist of two very different subpopulations—chains dominated by the faster-reacting monomer and chains containing long segments of the slower one. This produces much larger structural variability and, therefore, a high CV_GD_. In this way, CV_GD_ provides the second level of distinction, clearly separating random/alternating structures (low CV_GD_) from homopolymer-like ones (high CV_GD_), even though both are expected to be left skewed.

In blocky, less homopolymeric structures, most chains contain long contiguous segments of a single monomer. Introducing A/B toggle fragments into these segments produces intermediate block arrangements (e.g., diblock → triblock → tetrablock) without forming true gradient patterns. As the number of toggles increases, block segments become progressively fragmented, enhancing structural diversity within blocky chains, while nearly homopolymeric sequences remain comparably probable. These structural characteristics are reflected in positively skewed distributions ( ${\mathrm{\mu ̃}}_{3,GD}$ >0), where the tail extends toward higher GD values, indicating that chains with large GD occur with a higher probability relative to those near the average. Illustrations for ideal chain populations spanning blocky to random structures are provided in Section S3.1 of the Supporting Information.

Despite that ${\mathrm{\mu ̃}}_{3,GD}$ captures the asymmetry of the GD distribution and provides insight into the general arrangement of chains, it does not fully describe nongradient architectures. The coefficient of variation CV_GD_ complements this by quantifying the chain-to-chain consistency. Low CV_GD_ indicates that most chains closely resemble a single structural type, whether blocky, gradient-like, alternating, or random, whereas higher CV_GD_ reflects greater diversity. Alternating copolymers naturally produce low CV_GD_ due to their inherent symmetry, while blocky copolymers yield more asymmetric distributions and even homopolymer-like subpopulations, despite similar ⟨GD⟩ values.

In summary, ⟨GD⟩ is sufficient to distinguish gradient from nongradient copolymers, but the combined use of ${\mathrm{\mu ̃}}_{3,GD}$ and CV_GD_ is necessary to fully characterize nongradient architectures. Together, these metrics provide a comprehensive and quantitative framework for distinguishing alternating, random, blocky, and gradient sequences.

The structural deviation coefficient of variation (CV_SD_ = $\frac{\sigma_{SD}}{\left\langle SD\right\rangle }$ ) also provides interesting insights. The lowest CV_GD_ of 0.11 in Figure 5 is observed for the alternating P4 sample, for which the reactivity ratios favor cross-propagation, promoting balanced consumption of A and B and preventing long stretches (of any length) of a single monomer type (of any type). The random copolymer P3 is in turn characterized by an intermediate CV_GD_ value of 0.16. In both cases, the variability is minimized, due to intrinsic symmetry in monomer consumption. In contrast, gradient architectures introduce directional variability, with P1 exhibiting a higher CV_GD_ of 0.50. P2, the most structurally heterogeneous sample, has a CV_GD_ of 0.58, which is a result of its blocky character and subpopulations with divergent sequence patterns. In other words, transitions from monomer A to B inherently produce variable-length sequences composed solely of A or B, introducing additional variability in sequence length and structure.

## 3 Synthesis Parameters to Enhance the Gradient Quality

### 3.1 Importance of Reactivity Ratios in the Absence of Side Reactions

This subsection will discuss how the monomer structure influences the linear gradient quality of copolymers that are synthesized in silico through cationic ring opening polymerization (CROP) of 2-oxazolines. It is assumed for illustration purposes that the homopropagation rate coefficients are equal (*k*_pAA_ = *k*_pBB_, see Section S3.2 of the Supporting Information for cases *k*_pAA_ = 0.1*k*_pBB_ and *k*_pAA_ = 0.01*k*_pBB_), consistent with the sensitivity study by Conka et al.^47^ focusing on monomer sequence incorporation without the interference of side reactions.

More in detail, batch isothermal simulations are conducted with a target DP of 100, assuming perfect macroscale mixing in acetonitrile and complete monomer conversion to manifest the best achievable controlled “real” polymerization conditions. Symmetric initial comonomer amounts are employed, with the initiation rates set five times higher than homopropagation rates to fully enable ideal living polymerization characteristics. A comprehensive sensitivity analysis explores a wide kinetic parameter space by varying the monomer reactivity ratios (*r*_A_, *r*_B_) from 0.01 to 100 in silico, enabling a thorough evaluation of chemical reactivity that might yield the best possible high-quality gradient copolymers.

As shown in Figure 6 (*k*_pAA_ = *k*_pBB_), the analysis conclusively demonstrates that perfectly controlled gradient structures are unattainable, even in the absence of side reactions. Even in the most favorable region I (shown in subplot d but applicable to all subplots), in which reactivity ratios only slightly deviate from unity (*r*_A_ < 1, *r*_B_ > 1) achieving low ⟨GD⟩ (Figure 6a), the σ_GD_ is still only relatively low (Figure 6b), indicating unavoidable intermolecular variability in comonomer incorporation. This variability stems from the real-valued monomer inclusion probabilities inherent to gradient copolymers, which inevitably lead to a distribution of chain structures rather than uniform sequences. This is in contrast to block copolymers for which integer-valued probabilities can at least theoretically result in identical chains with zero σ_BD_.

**Figure 6:**
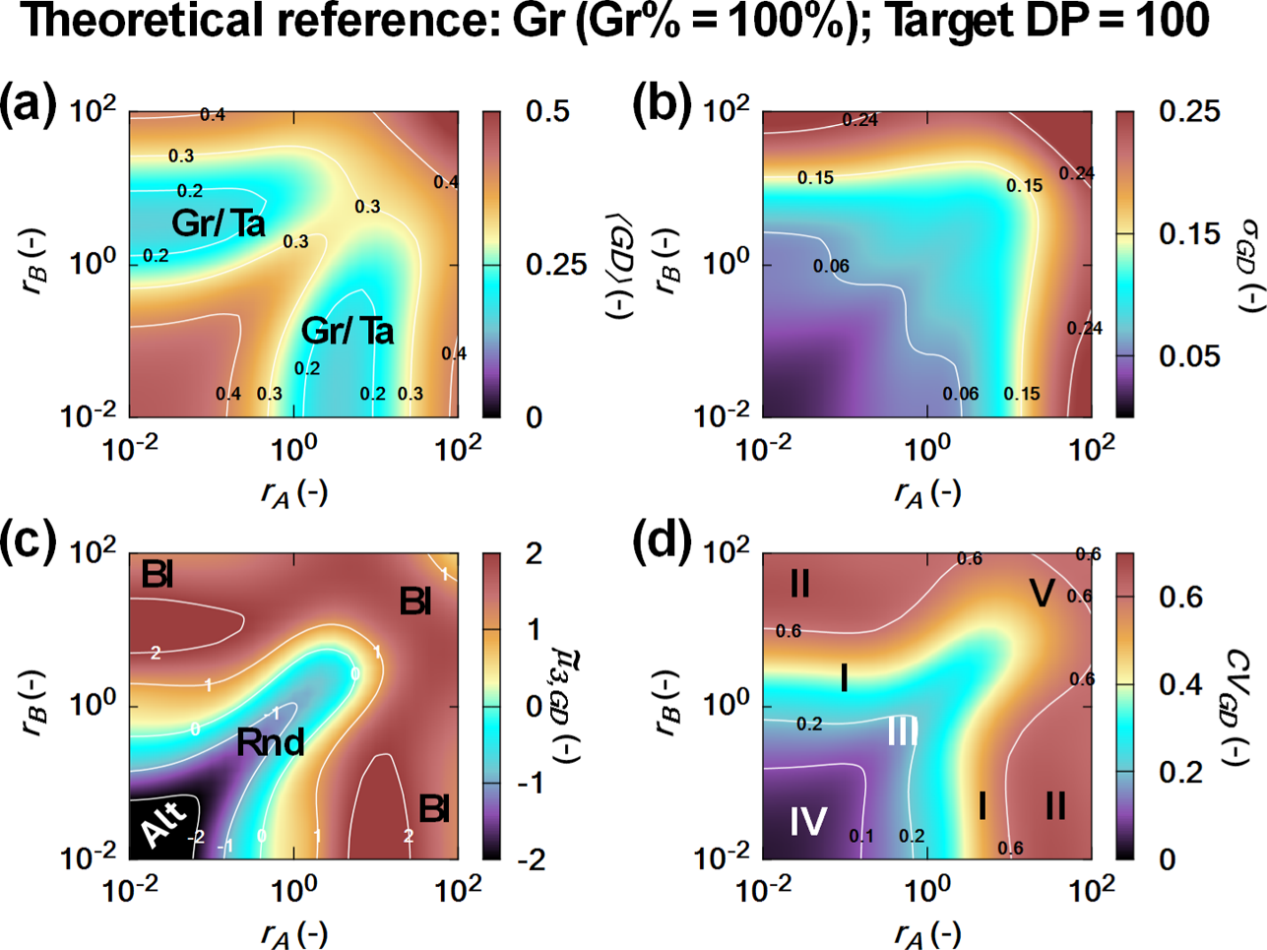
Effect of the monomer reactivity ratios (*r*_**A**_, *r*_B_) on the average gradient deviation ⟨GD⟩, gradient standard deviation (σ_GD_), gradient deviation skewness ${\mathrm{\mu ̃}}_{3,GD}$ , and gradient coefficient of variation (CV_**GD**_), assuming CROP without side reactions, equal homopropagation rate coefficients (*k*_pAA_ = *k*_pBB_), and with complete monomer conversion (target DP of 100). Labeling of the polymer type is also included. It follows that gradient (Gr) and tapered (Ta) copolymers exhibit low ⟨GD⟩, driven by balanced *r*_A_and *r*_B_ values that promote gradual monomer incorporation. In contrast, alternating (Alt; *r*_A_ ≈ *r*_B_≈0) or homopolymeric (HP)-driven (*r*_A_≫1, *r*_B_≪1 or vice versa) copolymers display high ⟨GD⟩ due to extreme reactivity ratios. Positive ${\mathrm{\mu ̃}}_{3,GD}$ values hold for copolymers with a compositional transition (gradient, tapered, or block). CV_GD_ values between 0.2 and 0.6 highlight significant intermolecular variability, indicating that highly controlled gradient structures are unattainable, due to a too pronounced inherent kinetic variation. Cases with different homopropagation rate coefficients: Figures S4 and S5 in the Supporting Information. Alternating and random sequences are visible in subplot (c), while homopolymer-like and gradient/tapered chains appear directly in subplot (a). This is because subplot (a) distinguishes very low GD (gradient) from very high GD (homopolymer), but intermediate GD values require subplot (c) to separate alternating/random (negative ${\mathrm{\mu ̃}}_{3,GD}$ , low CV_**GD**_) from blocky sequences (positive ${\mathrm{\mu ̃}}_{3,GD}$ , higher CV_GD_). Subplot (d) confirms the separation shown in subplot (c). Reprinted in adapted from with permission from R. Conka, Y. W. Marien, P. H. M. Van Steenberge, R. Hoogenboom and D. R. D’hooge, React. Chem. Eng., 2023, 8, 2905–2923. Copyright (2023) Royal Society of Chemistry.

In regions II–IV (shown in subplot d but applicable to all subplots), Figure 6a identifies where ⟨GD⟩ values become elevated. Figure 6c then clarifies the origin of these increases: positive ${\mathrm{\mu ̃}}_{3,GD}$ indicates blocky or homopolymer-like behavior (region II), whereas negative or near-zero skewness ${\mathrm{\mu ̃}}_{3,GD}$ reflects alternating or random character (region III–IV). These deviations arise from imbalanced cross-propagation kinetics that disrupt the desired gradient profile.

Simulations with unequal homopropagation rate coefficient (*k*_pAA_ ≠ *k*_*pBB*_) yield similar outcomes, leaning toward tapered structures but failing to eliminate compositional variability (see Figures S4 and S5 in the Supporting Information).

The consistent presence of nonzero σ_GD_ values in Figure 6b across all tested reactivity combinations with no side reactions highlights a fundamental boundary condition of ideal copolymerization kinetics, i.e., the stochastic nature of comonomer incorporation prevents the formation of perfectly controlled gradient copolymer chains, in which every chain exhibits an identical, smoothly transitioning compositional profile. This statement is also clear from the CV_GD_ data in Figure 6d, with values ranging between 0.2 and 0.6 highlighting (ideal) copolymer structures with significant compositional variation. Nonetheless, from Figure 6, it can be concluded that the most controlled gradient and tapered copolymer structures are obtained when one reactivity ratio is between 1 and 10 and the other is less than 1.

### 3.2 Importance of Side Reactions and Temperature

This subsection focuses on how side reactions and polymerization temperature variations affect the gradient quality for the CROP of 2-oxazolines as a model system, further increasing the realistic nature of the simulations compared to the previous section. An important factor is the ongoing competition for chain end functionality; any chain termination, transfer, or delayed initiation reduces or changes the number of active chain ends, directly limiting the ability to achieve predictable monomer sequences. Living or controlled polymerization conditions are, thus, essential for realizing gradient copolymer structures. Chain dispersity (*D̵*) plays a critical role in determining gradient quality. A high *D̵* broadens the distribution of chain compositions and reduces the fraction of chains that closely matches the target gradient. Hence, even under rather controlled polymerization conditions, achieving well-defined gradients requires careful optimization of synthesis parameters, such as semibatch addition protocols and minimization of side reactions, to limit dispersity variations and to maintain structural fidelity.

For illustration purposes, the copolymerization of 2-methyl-2-oxazoline and 2-phenyl-2-oxazoline (MeO*x* and PhO*x*)^60^ is selected as a case study, targeting gradient and block-gradient (tapered) copolymer structures. Two in silico copolymerization synthesis routes, namely a batch and semibatch one, are explored at 140 °C, with a target DP of 150, and 70% MeO*x* and 30% PhO*x* of overall composition. It should be noted that the kinetic parameters used have been validated with experimental data in previous work.^65^

The SD evaluation standard has been developed to provide quantitative and systematic measure of sequence structure quality.^27^ For symmetric compositions, the excellent/good threshold (=0.06) could be analytically determined as the average of the target copolymer distribution. The good/poor threshold (=0.3) is chosen more arbitrarily as a practical reference for target DP 100 but supported by inspection of plots of explicit monomer sequences. For asymmetric compositions (70/30) with target DP 150, these thresholds were adapted accordingly (⟨SD_Exc/Good_⟩ = 0.05; ⟨SD_Good/Poor_⟩ = 0.1),^60^ recognizing that the distribution statistics differ from the symmetric case. A full analytical framework was established later,^25^ for which the details are provided in Section S3.3.3 of the Supporting Information. In Figure 7a, the color coding reflects this classification: excellent quality chains (green background) closely follow the ideal sequence, good chain quality chains (blue background) deviate moderately, and chains of poor quality (gray background) deviate strongly.

**Figure 7:**
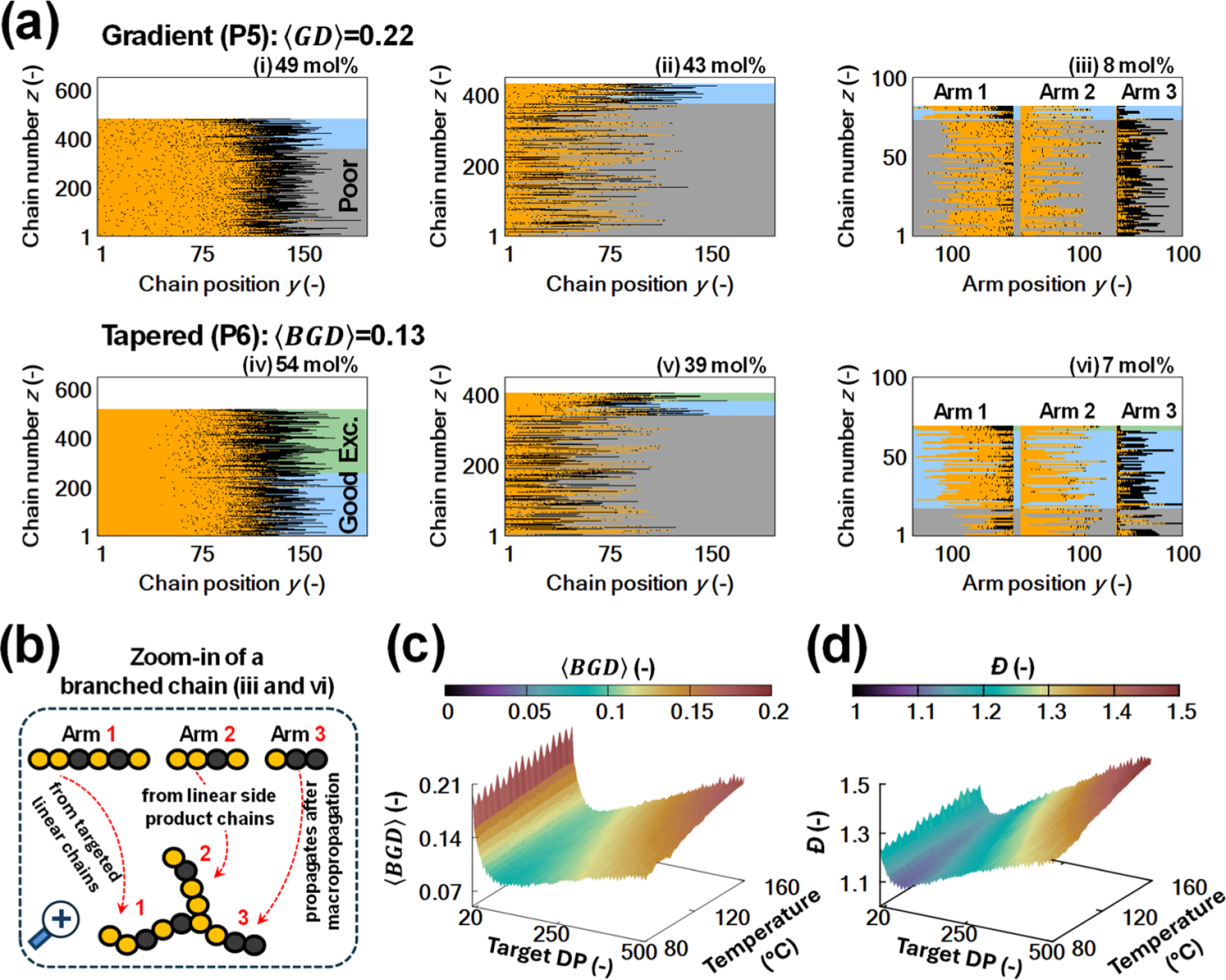
(a) Effect of side reactions aiming at gradient (top) and tapered (bottom) copolymer compositions via in silico CROP in acetonitrile at 140 °C and a target DP of 150, considering an overall A:B composition of 105:45; left column: no side reactions; middle column: with also chain transfer to monomer; and right column: with also macropropagation (all reactions; Section S3.2 of the Supporting Information). For the right column, single branched chains are plotted as 3 arms next to each other. Background color coding: green: excellent quality: ⟨SD⟩ ≤ 0.05; blue: good quality: 0.05 < ⟨SD⟩ ≤ 0.1; gray: bad quality: ⟨SD⟩ > 0.1; model parameters from ref 60 (b) A zoom-in of a branched chain, as shown in (iii) and (vi). (c,d) Effect of the target degree of polymerization (target DP) and the polymerization temperature for semibatch synthesis procedures with monomers MeO*x* and PhO*x* (always 70:30 final composition; full reaction scheme and recipes; Section S3.2 of the Supporting Information), aiming at a tapered structure: (c) ⟨BGD⟩ and (d) the dispersity at 0.98 overall monomer conversion. ⟨SD⟩ labels for all reactions case, calculated as follows: for P5, the average is taken over cases (i–iii); for P6, the average is taken over cases (iv–vi). Mol % refers to the contribution of targeted linear product (P5:i; P6: iv), linear side product (P5: ii; P6: v), and branched side product (P5: iii; P6: vi). The SD metrics can directly be interpreted as GD for P5 and BGD for P6. Reprinted in adapted from with permission from R. Conka, Y. W. Marien, O. Sedlacek, R. Hoogenboom, P. H. M. Van Steenberge and D. R. D’hooge, Polym. Chem., 2022, 13, 1559–1575. Copyright (2022) Royal Society of Chemistry.

Figure 7a provides a snapshot of the compositional variations (top: gradient target; bottom: tapered target), highlighting from left to right the influence of more side reactions on the compositional quality, with first no side reactions, then chain transfer to monomer included, and then also macropropagation. The *P*_A/B_ profiles, the targeted structures, the synthesis details, and a CMMC flowsheet along with the interpretation of matrix representations for linear and branched species are given in Section S3.3.4 of the Supporting Information.

The batch synthesis route generates P­(MeO*x*_150_-*grad*-PhO*x*_45_), denoted as P5. In contrast, the semibatch route initiates polymerization with MeO*x* alone and introduces only PhO*x* after a MeO*x* conversion of 0.60, producing P­(MeO*x*_60_-block-(MeO*x*_45_-*grad*-PhO*x*_45_)), labeled P6. The structural quality of these copolymers has been evaluated by comparing P5 and P6 against their respective gradient and tapered targets, explaining the calculation of ⟨GD⟩ and ⟨BGD⟩ (see top lines of Figure 7a). Note that panels (i) and (iv) show the targeted linear products for P5 and P6, panels (ii) and (v) show the linear side products for P5 and P6, and panels (iii) and (vi) show the branched side products for P5 and P6 (Figure 7b shows how branched chains are represented). It is important to note that while P5 and P6 have the same overall composition (70/30 MeO*x*/PhO*x*, with target DP 150), they are prepared using different synthesis strategies targeting distinct structures: a gradient for P5 and a block-gradient (tapered) structure for P6. Accordingly, the SD metrics are selected to reflect these intended targets (GD for P5, BGD for P6; see Section S3.4.2 of the Supporting Information). Direct comparison of the absolute SD values between P5 and P6 is therefore not meaningful as the metrics correspond to different target structures. Nevertheless, the methodology is consistent across both polymers: each SD metric evaluates how well the respective synthesis strategy achieves its predefined target, allowing for a fair assessment of structural fidelity within each design framework. In general, for a given synthesis recipe, the in silico tool can identify the structure that could be seen as the best target, in that way justifying the calculation of a series of SD parameters.

In the (overall) conversion range of 0.70–1.0, in which PhO*x* incorporation becomes prominent,^60^ chain transfer to monomer disrupts the targeted structure by promoting excess PhO*x* incorporation in shorter chains. This effect is more pronounced in the batch process (P5), in which both MeO*x* and PhO*x* are present from the start, leading to higher rates of chain transfer to monomer due to greater monomer availability and comparable chain transfer rate coefficients for MeO*x* and PhO*x*. P5 exhibits therefore a higher molar fraction of defective linear chains when chain transfer to monomer is added as a reaction possibility. The middle column in Figure 7a depicts 43 mol % of such chains in P5 compared to 39 mol % in P6, resulting in elevated average structural deviation: ⟨SD⟩ = 0.22 for P5 versus 0.13 for P6. The semibatch strategy in P6 mitigates these chain transfer to monomer side reactions by delaying PhO*x* addition, reducing the opportunity for chain transfer and thereby decreasing the formation of defective chains. This leads to improved structural quality, as evidenced by the lower SD values.

Furthermore, in Figure 7a, the background of the chains is colored according to their quality; for excellent compositions, ⟨SD⟩ ≤ 0.05 (green), for good compositions, 0.05 < ⟨SD⟩ ≤ 0.15 (blue), and for poor compositions, ⟨SD⟩ > 0.15 (gray). The semibatch product P6 shows a higher proportion of excellent chains, reflecting again an improved structural fidelity achieved through a reduced importance of side reactions. In contrast, P5 contains more poor-quality chains, a consequence of increased chain transfer to monomer, leading to a higher compositional dispersity and diminished compositional accuracy.

It should be noted that the synthesis of tapered (block-gradient) MeO*x*/PhO*x* copolymers such as P6 involves a more intricate semibatch process compared to the simpler batch route for gradient copolymers such as P5. The semibatch route requires more dedicated and elaborate synthetic procedures, starting with MeO*x* polymerization followed by timed PhO*x* addition at a given MeO*x* conversion of 0.60, making it more complex to execute and follow-up for production quality control. Despite these challenges, the effort is worthwhile, as the tapered structure (P6) achieves superior structural quality with lower average structural deviation and fewer defective chains, as discussed in Figure 7a.

The reaction temperature and target DP are additional variables, and their effect on block gradient copolymer quality is depicted in Figure 7c for the semibatch strategy, considering a temperature range of 80–160 °C and target DPs from 10 to 500. This grid simulation reveals a constrained window for achieving optimal ⟨BGD⟩, with the best outcomes limited to specific temperature-DP combinations. Notably, as shown in Figure 7d, the dispersity (*D̵*) tracks a similar trend as the ⟨BGD⟩ profile, although the experimental determination of this molecular property can be less straightforward for copolymer systems. Hence, it is worthwhile to enrich the characterization toolbox with in silico methods.

## 4 Perspectives

According to fundamental kinetic principles, the ambition of synthesizing perfectly controlled gradient copolymers via RDRP or LP techniques, in which each chain mirrors an identical, seamlessly transitioning A-to-B compositional profile, is unrealistic. This is already clear from calculating a set of so-called structural deviation (SD) metrics for an ideal copolymer structure with chains of equal length and a composition following targeted monomer inclusion probabilities. Specifically, for targeted structures with a higher gradient character, the nonzero standard deviation (σ_SD_) is more evident, and the coefficient of variation (CV_SD_) is moderate, with values between 0.2 and 0.6. Gradient and tapered copolymers inherently allow for variable segment lengths of each comonomer type, which broadens the compositional distribution and elevates CV_SD_.

Under realistic polymerization conditions, with side reactions such as chain transfer to monomer and macropropagation for the selected illustrative CROP chemistry, additional molecular deviations are expected. However, by proper design of the monomer structure and reaction conditions, a maximal molecular control is within reach. For instance, semibatch procedures can be worthwhile as well as branch formation in which structural imbalances on monomer incorporation can be rectified.

Notably, the increase in copolymer (e.g., gradient) quality can be quantified by running CMMC simulations in parallel with experimental programs. At any stage of the experimental protocol development and design, a CMMC simulation can be performed to check if a given target will be more easy to reach or not, provided that some reference homo- and copolymerization experiments have been conducted to tune the most essential kinetic parameters. The advantage of CMMC models is that the experimental validation needed can be provided with more experimental tools as accessible by the typical polymer chemist, opening the door to new software integrations in existing analytical tools on the longer run.

The current perspective thus transforms an inherent constraint on compositional variability into a design opportunity. By bridging advanced modeling and tailored synthesis, in silico exploiting less common mitigation strategies, it will be more facile to design gradient copolymers with predictable properties, such as customized mechanical, thermal, or responsive behaviors.

Recognizing that perfectly controlled gradients are unachievable inspires a forward-looking approach, driving innovation in copolymer design that balances structural diversity with functional precision, positioning gradient copolymerization as a vibrant field for engineering next-generation materials within the realistic bounds of polymerization kinetics and physics. In this context, it should be noted that properties and self-assembly behavior of polymeric materials can also benefit from structural heterogeneity,^66,67^ further indicating the importance of gradient and tapered copolymers.

## Supplementary Material



## Data Availability

The data are available in the open literature and its Supporting Information.

## Notes

Calculation of (average) structural deviation, model details, and additional simulation results (PDF)
