# Supplementary material for: Gradient Copolymers: A Complex Comonomer Incorporation Reality behind the Perfect Ideal
Source: ACS Polym Au. 2026 Mar 6;6(2):506–19. doi: 10.1021/acspolymersau.5c00147 (PMC13067163; doi:10.1021/acspolymersau.5c00147)
Supplement: Supplementary file 1 [file lg5c00147_si_001.pdf]

Supporting Information for "Gradient copolymers: a complex comonomer incorporation reality behind the perfect ideal"

Robert Conka,[1] Yoshi W. Marien,[1,3] Kevin M. Van Geem,[1] Paul H.M. Van Steenberge,[1] Richard Hoogenboom,[2*] Dagmar R. D'hooge[1,4*]

[1] Laboratory for Chemical Technology (LCT), Ghent University, Technologiepark 125, 9052 Ghent, Belgium

[2] Supramolecular Chemistry Group, Centre of Macromolecular Chemistry (CMaC), Department of Organic and Macromolecular Chemistry, Ghent University, Krijgslaan 281-S4, 9000 Ghent, Belgium

[3] Intelligence in Processes, Advanced Catalysts and Solvents (iPRACS), Faculty of Applied Engineering, University of Antwerp, Groenenborgerlaan 171, 2020 Antwerp, Belgium

[4] Centre for Textiles Science and Engineering (CTSE), Ghent University, Technologiepark 70a, 9052 Ghent, Belgium

Corresponding author:

Prof. Dr. Richard Hoogenboom; E-mail: richard.hoogenboom@ugent.be

Prof. Dr. Dagmar R. D'hooge; E-mail: dagmar.dhooge@ugent.be

TABLE OF CONTENTS

S1 Calculation of (Average) Structural Deviation 3
S1.1 Step 1: Monomer Inclusion Probabilities 3
S1.1.1 Symmetric copolymer compositions 5
S1.1.2 Asymmetric copolymer compositions 7
S1.2 Step 2: Generation of Ideal Linear Polymer Samples and $\boldsymbol{SD}/\boldsymbol{SD}$ calculation 11
S1.3 Step 3: Normalization 12
S1.4 Summary of previous steps as workflow 12
S2 Model details 13
S2.1 Cationic ring opening polymerization (CROP) 15
S3 Extra simulation results 17
S3.1 Characterizing copolymer structures through $\boldsymbol{GD}$ distribution shape 17
S3.2 Reference simulations 17
S3.3 Effect of reactivity ratios (no side reactions) 18
S3.4 Effect of side reactions and polymerization temperature 20
S3.4.1 Cationic ring opening polymerization (with side reactions) 20
S3.4.2 Targeted structures 22
S3.4.3 SD evaluation standard 23
S3.4.4 Branched chain algortihm 26
S4 References 27

S1 CALCULATION OF (AVERAGE) STRUCTURAL DEVIATION

The calculation of structural deviation for a chain (SD) and its average value ($\langle SD \rangle$) relies on a set of core mathematical principles previously developed to quantify how closely a polymer chain aligns with a predefined target structure. These methods were initially formulated for symmetric (linear) gradient copolymers by Van Steenberge *et al.*[1] and further refined by Fierens *et al.*[2] Later, Toloza *et al.*[3] extended the approach to symmetric diblock copolymers, and Conka *et al.*[4, 5] generalized the framework to include asymmetric target structures, allowing for a unified treatment across a wide range of copolymer architectures.

In what follows, the main steps of the aforementioned mathematical principles will be discussed.

S1.1 STEP 1: MONOMER INCLUSION PROBABILITIES

The monomer inclusion probabilities for comonomers A (P_A) and B (P_B) are defined under two constraints: (1) at any chain position y, the probabilities sum to unity, *i.e.* $P_A(y) + P_B(y) = 1$, and (2) their integrated contributions across the full chain length ω match the initial monomer feed ratios, $x_{A,0}$ and $x_{B,0}$, respectively.

To describe the shape of the target structure, two key parameters have been introduced by Conka *et al.*[5] The gradient fraction $f_{Gr,Sym/Asym}$ denotes the proportion of the chain exhibiting a linear gradient transition from A to B, while the block fraction $f_{Bl,Sym/Asym}$ represents the fraction with block-like features. By definition, $f_{Gr,Sym/Asym} + f_{Bl,Sym/Asym} = 1$. Adjusting these parameters allows precise control over the gradient steepness in the designed copolymer architecture.

Monomer inclusion probabilities are constructed as functions within a two-dimensional Cartesian coordinate system, where the x-axis corresponds to chain position y and the y-axis to the probability

$P_{A/B}(y)$. The construction of the B monomer inclusion probability $P_B(y)$ is first detailed for symmetric targets for which $x_{A,0} = x_{B,0} = 0.5$ and then extended to asymmetric targets where $x_{A,0} > x_{B,0} < 0.5$.

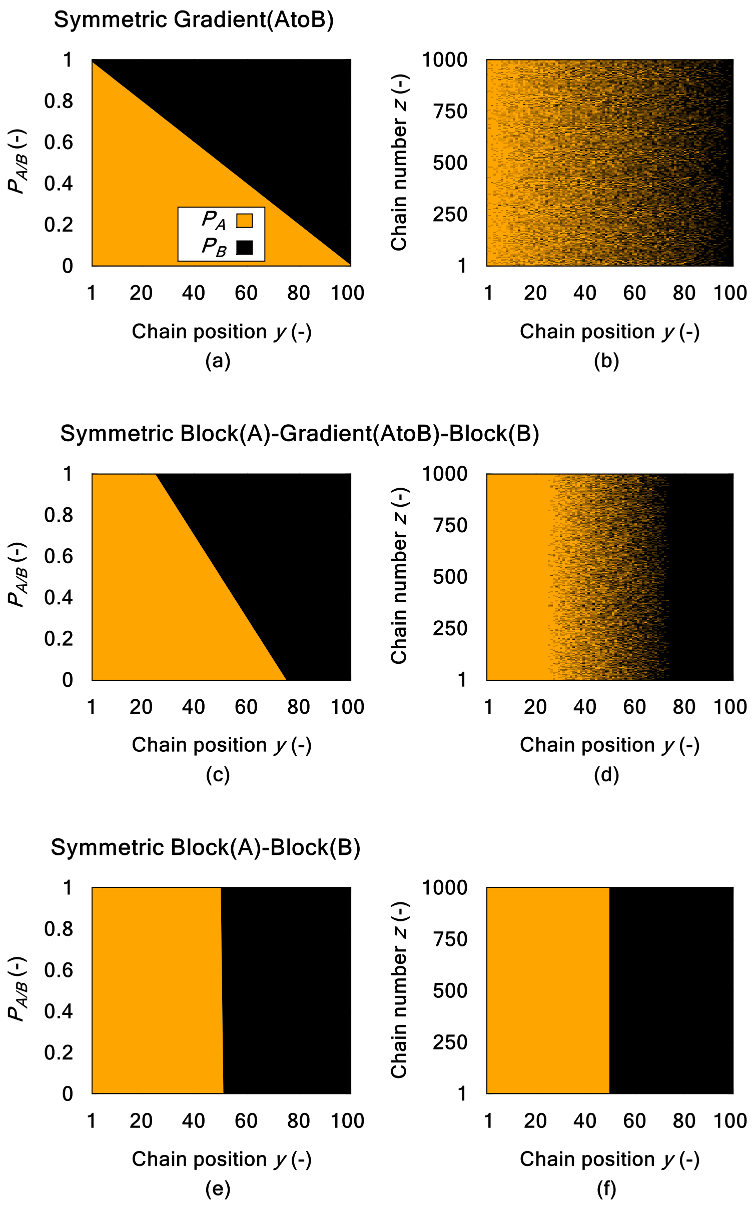

Figure S1. Left column: the monomer inclusion probability equations shown row stacked with P_A (dark-blue) and P_B (light-green) for the symmetric targets (see also Table 1). Right column: the corresponding ideal linear polymer samples of target DP 100: (a,b) $f_{Gr,Sym}$ = 1; (c,d) $f_{Gr,Sym}$ = 0.5; (e,f) $f_{Gr,Sym}$ = 0.

S1.1.1 Symmetric copolymer compositions

For symmetric copolymers exhibiting a linear shift from monomer A to B, the boundary conditions $P_B(y = 1) = 0$ and $P_B(y = \omega) = 1$ hold. These endpoints enable a straightforward construction of the mathematical expression describing the P_B profile.

S1.1.1.1 Symmetric 100% Gradient copolymers

For a symmetric copolymer with a fully (100%) gradient, defined by $f_{Gr,Sym} = 1$ and $f_{Bl,Sym} = 0$, each chain position $y \in [2, \omega - 1]$ is associated with a P_B value that takes a continuous value in the open interval $(0,1)$, or $P_B(1 < y \leq \omega - 1) \in]0,1[$. By constructing a linear relationship that satisfies the boundary conditions $P_B(1) = 0$ and $P_B(\omega) = 1$, the following expression is obtained:

$$P_B\left(f_{Gr,Sym} = 1; 1 \leq y \leq \omega\right) = \frac{y - 1}{\omega - 1} \qquad (S1)$$

Figure S1 illustrates this monomer inclusion profile, where subplot (a) shows the linear progression of P_B across the chain, and subplot (b) displays the corresponding ideal polymer structure for a 50:50 A:B copolymer with a target degree of polymerization (DP) of 100.

S1.1.1.2 Symmetric f_{Gr}, f_{Bl} $\in]\mathbf{0}, \mathbf{1}[$ copolymers

The parameter $f_{Gr/Bl,Sym} \in]0,1[$ defines copolymers with a block-gradient-block structure. Low values of $f_{Gr,Sym}$ correspond to steep gradient transitions within the copolymer structure. The construction of such a sequence involves three distinct regions.

Table S1. Description of the three sequential regions comprising an ideal symmetric Block–Gradient–Block copolymer structure. Each region is defined by its length (as a fraction of the total chain length ω) and the corresponding probability P_B of incorporating monomer B.

A-block (start)	A sequence of length $\frac{f_{Bl,Sym}}{2}\omega$ composed entirely of monomer A, where the monomer B inclusion probability is P_B=0 (see Eq. S2).
Gradient region	A linear gradient segment of length $f_{Gr,Sym}\omega$ where or $P_B \in]0,1[$ and the gradient satisfies $\frac{dP_B}{dy} > 0$, representing a progressive transition from A to B (see Eq. S3).
B-block (end)	A final segment of length $\frac{f_{Bl,Sym}}{2}\omega$ containing only monomer B, for which P_B=1 (see Eq. S4).

These regions can each be described using distinct mathematical expressions that define the profile of $P_B(y)$ across the chain:

$$P_B\left(0 \le f_{Gr,Sym} \le 1; \frac{f_{Bl,Sym}}{2}\omega + f_{Gr,Sym}\omega < y \le \omega\right) = 1 \tag{S2}$$

$$P_B\left(0 \le f_{Gr,Sym} \le 1; \frac{f_{Bl,Sym}}{2}\omega < y \le \frac{f_{Bl,Sym}}{2}\omega + f_{Gr,Sym}\omega\right) = \frac{y - \frac{f_{Bl,Sym}}{2}\omega}{f_{Gr,Sym}\omega} \tag{S3}$$

$$P_B\left(0 \le f_{Gr,Sym} \le 1; \frac{f_{Bl,Sym}}{2}\omega + f_{Gr,Sym}\omega < y \le \omega\right) = 1 \tag{S4}$$

Figure S1 illustrates the monomer B inclusion profile P_B (subplot **(c)**) alongside the corresponding ideal polymer sequence (subplot **(d)**) for a symmetric 50:50 A:B copolymer with a target DP 100 and and $f_{Gr,Sym} = 0.5$.

S1.1.1.3 Symmetric 100% Di-block copolymers

In the specific case of a 100% block copolymer, *i.e.* $f_{Gr,Sym} = 0$ and $f_{Bl,Sym} = 1$, any chain position $y \in \left[1, \frac{\omega}{2}\right]$ is characterized by an integer number zero for P_B, while $y \in \left[\frac{\omega}{2} + 1, \omega\right]$ is characterized by an

integer number one for P_B. This discrete switch in values at the chain's midpoint allows the definition of a set of block-functions shown in Eq. S5 (block A*) and S6 (block B): [3]

$$P_B\left(f_{Bl} = 1; 1 \leq y \leq \frac{\omega}{2}\right) = 0 \tag{S5}$$

$$P_B\left(f_{Bl} = 1; \frac{\omega}{2} < y \leq \omega\right) = 1 \tag{S6}$$

In **Figure S1**, the P_B-profile (subplot **(e)**) and the corresponding ideal linear polymer sample (subplot **(f)**) are shown for a 50:50 A:B copolymer of target DP 100, and $f_{Gr,Sym} = 0$.

S1.1.2 Asymmetric copolymer compositions

In contrast to symmetric copolymers, in which there is a clear transition from block A to block B, characterized by $P_B(y = 1) = 0$ and $P_B(y = \omega) = 1$, asymmetric systems behave differently. While the initial condition $P_B(y = 1) = 0$ still applies, the final position at $y = \omega$ cannot be assumed to reach $P_B = 1$. Instead, due to the unequal starting quantities of monomers A and B, the terminal value satisfies only $P_B(y = \omega) < 1$. In the extreme scenario in which no B units are initially present, we arrive at $P_B(y = \omega) = 0$, which corresponds to a pure A homopolymer, outside the scope of relevant copolymer structures considered here.

S1.1.2.1 Asymmetric 100% Gradient copolymers

In the case of a 100% gradient copolymer, *i.e.* $f_{Gr,Asym} = 0$ and $f_{Bl,Asym} = 1$, a linear equation can be found:

$$P_B(f_{Gr} = 1; 0 < y \leq \omega) = \frac{2x_{B,0}(y-1)}{\omega - 1} \tag{S7}$$

Figure S2 displays the P_B distribution in subplot (**a**), alongside the corresponding idealized linear polymer configuration in subplot (**b**), for a 70:30 A:B copolymer with a target degree of polymerization (DP) of 100 and a symmetry fraction $f_{Gr,Sym} = 1$.

S1.1.2.2 Asymmetric f_{Gr}, f_{Bl} ∈ $]\mathbf{0},\mathbf{1}[$ copolymers

As previously noted, asymmetric copolymers are characterized by the condition $P_B(y=\omega)<1$. This naturally gives rise to two types of copolymers with $f_{Gr/Bl,Asym}$ ∈ $]0,1[$. The first class consists of Block(A)-Gradient copolymers, where $f_{Bl,Asym}$ is relatively low, resulting in a gradual transition from A to B. The second type features a more complex architecture: Block(A)-Gradient(AtoB)-Block(B), corresponding to higher values of $f_{Bl,Asym}$, in which the gradient region is flanked by more clearly defined A and B blocks.

S1.1.2.2.1 Asymmetric Block(A)-Gradient copolymers

Upon considering an asymmetric copolymer with a relatively long linear gradient segment, the molecular architecture can be divided into two distinct regions. The initial portion forms a Block(A) domain, followed by a segment in which the composition transitions linearly from A to B. Due to the early consumption of monomer A within the Block(A) region, the subsequent gradient section exhibits a reduced mole fraction of A ($x_{A,Gr}$) compared to its initial value ($x_{A,0}$). Consequently, the mole fraction of B in the gradient region ($x_{B,Gr}$) exceeds the initial B fraction ($x_{B,0}$). The quantitative relationship between the initial B monomer content and its specific concentration within the gradient region is described by Eq. S8:

$$x_{B,0}=f_{Gr,Asym}\cdot x_{B,Gr} \qquad (S8)$$

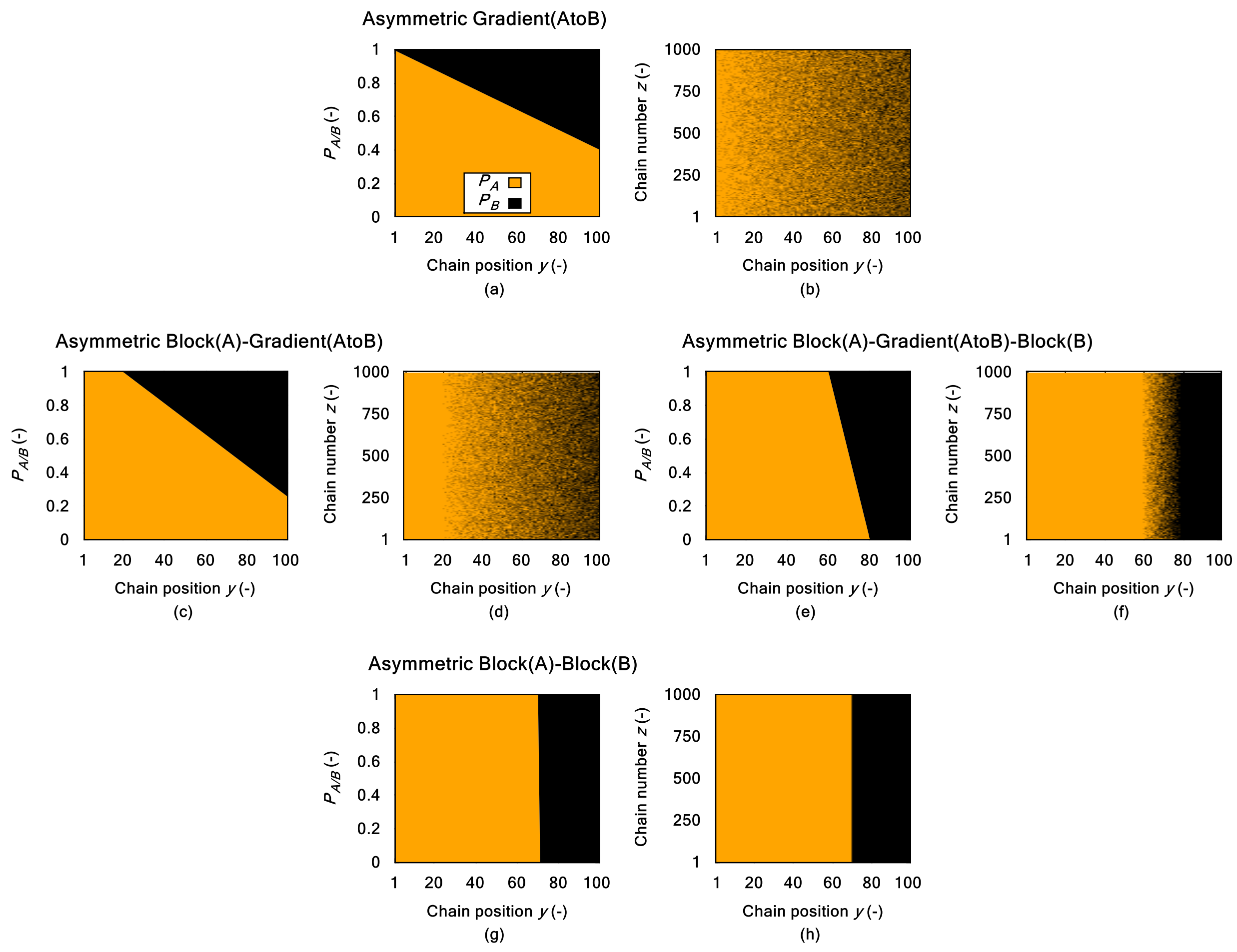

Figure S2. The monomer inclusion probability equations shown row stacked with P_A (dark-blue) and P_B (light-green) for the asymmetric targets (see also Table 2) and the corresponding ideal linear polymer samples of target DP 100: (a,b) $f_{Gr,Asym}$ = 1; (c,d) $f_{Gr,Asym}$ = 0.8; (e,f) $f_{Gr,Asym}$ = 0.2; (g,h) $f_{Gr,Asym}$ = 0.

The minimum required gradient sequence fraction, denoted $f_{Gr,Asym,Min}$, defines the shortest gradient length necessary to construct valid asymmetric Block(A)-Gradient copolymers. This threshold is given by the following expression:

$$f_{Gr,Min} = 2x_{B,0} \qquad (S9)$$

The P_B-equation for an asymmetric block(A)-gradient(AtoB) is given by:

$$P_B\left(f_{Gr,Asym,Min} \leq f_{Gr} < 1; 1 \leq y \leq f_{Bl,Asym} \cdot \omega\right) = 0 \qquad (S10)$$

$$P_B\left(f_{Gr,Asym,Min} \leq f_{Gr,Asym} < 1; f_{Bl,Asym} \cdot \omega < y \leq \omega\right) = \frac{2x_{B,Gr}\left(y - f_{Bl,Asym}\omega\right)}{\omega - f_{Bl,Asym}\omega} \quad (S11)$$

Figure S2 presents the P_B-profile in subplot (c), alongside the corresponding ideal linear polymer sample in subplot (d), for a 70:30 A:B copolymer of target DP 100, and $f_{Gr,Sym} = 0.8$.

S1.1.2.2.2 Asymmetric BlockA-Gradient-BlockB copolymers

For asymmetric copolymers in which $f_{Gr,Asym} < f_{Gr,Asym,Min}$, the composition profile follows the form of an asymmetric block(A)-gradient(AtoB)-block(B) structure. The corresponding P_B expression is given by:

$$P_B\left(f_{Gr,Asym} < f_{Gr,Asym,Min}; 1 \leq y \leq \left(x_{A,0} - \frac{f_{Gr,Asym}}{2}\right) \cdot \omega\right) = 0 \quad (S12)$$

$$P_B\left(f_{Gr,Asym} < f_{Gr,Asym,Min}; \left(x_{A,0} - \frac{f_{Gr,Asym}}{2}\right) \cdot \omega < y \leq \left(x_{A,0} + \frac{f_{Gr,Asym}}{2}\right) \cdot \omega\right) = \frac{y - x_{B,0}\omega + \frac{f_{Gr,Asym}\omega}{2}}{f_{Gr,Asym}\omega} \quad (S13)$$

$$P_B\left(f_{Gr,Asym} < f_{Gr,Asym,Min}; \left(x_{A,0} - \frac{f_{Gr,Asym}}{2}\right) \cdot \omega < y \leq \omega\right) = 1 \quad (S14)$$

Figure S2 illustrates the P_B-profile in subplot **(e)**, alongside the corresponding ideal linear polymer sample shown in subplot **(f)**, for a 70:30 A:B copolymer with a target DP 100 and $f_{Gr,Sym} = 0.2$.

S1.2.2.3 Asymmetric 100% Di-block copolymers

Similar to symmetric copolymers, for any chain position $y \in [1, x_{A,0}\omega]$, the value of P_B is assigned as zero, indicating the presence of monomer A. For positions $y \in [x_{A,0}\omega + 1, \omega]$, P_B takes the value one, representing monomer B. This stepwise behavior is captured by the following stepwise equation:[6]

$$P_B\left(f_{Bl} = 1; 1 \leq y \leq x_{A,0}\omega\right) = 0 \quad (S15)$$

$$P_B\left(f_{Bl} = 1; x_{A,0}\omega < y \leq \omega\right) = 1 \quad (S16)$$

Figure S2 presents the P_B-profile in subplot **(g)** alongside the corresponding ideal linear polymer sample shown in subplot **(h)**, for a 70:30 A:B copolymer with a target DP 100 and $f_{Gr,Sym} = 0$.

S1.2 STEP 2: GENERATION OF IDEAL LINEAR POLYMER SAMPLES AND $SD/\langle SD \rangle$ CALCULATION

For each chain z in an ideal linear polymer sample, the cumulative amounts of comonomers A and B at every chain position y, denoted $S_{A/B}(y,z)$,[1] can be directly compared to the theoretical cumulative values expected for the target polymer structure.[1]

$$S_{A/B,ideal}(y) = \sum_{y=1}^{\omega} P_{A/B}(y) \tag{S17}$$

As the $P_{A/B}(y)$ profiles generally do not consist of exact integers, except in special cases in which incorporation is certain ($P_{A/B}(y) = 1$) or completely absent ($P_{A/B}(y) = 0$), assigning chain positions y to specific comonomer types based on these probabilities is inherently a stochastic process. As a result, ideal linear polymer samples may exhibit discrepancies in the placement of A or B units relative to the intended $P_{A/B}(y)$ profile. These mismatches are quantified by the absolute structural deviation, denoted as SD^*.

As detailed in previous work,[1] the value of this parameter is determined by selecting the minimum from a set of structural deviation values: four in the case of symmetric copolymers and two for asymmetric ones. These values represent different possible mappings of the actual sequence onto the target profile. Of particular importance is the "left to right" evaluation, which is computed as follows:

$$SD_{Ideal} ** (y=\omega,z) = \sum_{y=1}^{\omega} \frac{1}{2} \frac{\left|S_{A,ideal}(y) - S_{A,Theor}(y,z)\right| + \left|S_{B,ideal}(y) - S_{B,Theor}(y,z)\right|}{\omega^2} \tag{S18}$$

However, the sequence of a copolymer that is read from "left to right" may differ from that read "right to left", especially in asymmetric systems. To account for this, an additional subscript (′) is introduced to distinguish between the two orientations. As described in earlier work, the final structural deviation for a given chain z of length ω is practically defined as:

$$SD^*_{Ideal}(z) = min\{SD_{Ideal} ** (y=\omega,z), SD'_{Ideal} ** (y=\omega,z)\} \tag{S19}$$

In practice, the goal is to determine an average structural deviation by summing all individual $SD^*_{Ideal}(z)$ values and dividing by z_{max}, the total number of polymer chains in the sample:

$$\langle SD^*_{Ideal} \rangle = \sum_{z=1}^{z_{max}} \frac{SD^*_{Ideal}(z)}{z_{max}} \tag{S20}$$

S1.3 Step 3: Normalization

As emphasized by Conka *et al.*,[4] in both symmetric and asymmetric cases, the homopolymer reference value $\langle SD^*_{HP} \rangle$ should be evaluated from right to left in the case of a B-homopolymer, representing the worst-case scenario for structural deviation:

$$\langle SD^*_{HP} \rangle = \langle SD'^*_{HP,B} \rangle \tag{S21}$$

The average structural deviation $\langle SD^* \rangle$ can be normalized by dividing it by the corresponding homopolymer reference value $\langle SD^*_{HP} \rangle$, yielding a rescaled measure of structural deviation:

$$\langle SD \rangle = \frac{\langle SD^* \rangle}{\langle SD^*_{HP} \rangle} \tag{S22}$$

This way, the $\langle SD \rangle$ has a value between 0 and 1. The value 0 can only be achieved for perfectly non-erroneous incorporations of A and B according to the $P_{A/B}$ profiles, and this for every chain and for every position, which is only possible for A-homopolymer and di-block targets but not for gradient(-dominated) targets. On the other hand, a $\langle SD \rangle$ value of 1 indicates that the evaluated polymer is a B-homopolymer.

S1.4 Summary of previous steps as workflow

In this form, the rescaled average structural deviation $\langle SD \rangle$ ranges between 0 and 1. A value of 0 signifies perfect fidelity in the incorporation of A and B monomers according to the target $P_{A/B}$ profiles, and this across all chains and all positions. Such ideal case is only attainable in specific scenarios, such as A-homopolymers or perfectly formed block copolymers. Conversely, a value of 1 indicates that the evaluated

polymer behaves as a B-homopolymer, representing the maximum possible deviation from the intended structure. **Table S2** provides an overview of the SD framework for gradient and block-type architectures.

Table S2. Summary of the structural deviation (SD) framework applied to gradient and block-type architectures. The table specifies the theoretical target profiles $S_{A/B,Theor}$, the absolute deviation metrics (GD^*, $BGBD^*$, BGD^*, BD^*) for both ideal and kinetically generated samples, and their normalized forms obtained by dividing by the respective homopolymer limits. Each metric quantifies the deviation of a given sample from its corresponding theoretical gradient, block-gradient-block, block-gradient, or block reference.

Input molecule	Metric	Gradient (Gr)	Block-Gradient-Block (Ta)	Block-Gradient (Ta)	Block (Bl)	Chain length
Theoretical reference molecule	$S_{A/B,Theor}$	$S_{A/B,Theor,Gr}$	$S_{A/B,Theor,BlGrBl}$	$S_{A/B,Theor,BlGr}$	$S_{A/B,Theor,Gr}$	Unique Chain Length (UCL)
	$SD^*_{HP,Theor}$	GD^*_{HP} (y vs. $S_{A/B,Theor,Gr}$)	$BGBD^*_{HP}$ (y vs. $S_{A/B,Theor,BlGrBl}$)	BGD^*_{HP} (y vs. $S_{A/B,Theor,BlGr}$)	BD^*_{HP} (y vs. $S_{A/B,Theor,Gr}$)	
Ideal sample	SD^*_{Ideal}	GD^*_{Ideal} ($S_{A/B,Ideal}$ vs. $S_{A/B,Theor,Gr}$)	$BGBD^*_{Ideal}$ ($S_{A/B,Ideal}$ vs. $S_{A/B,Theor,BlGrBl}$)	BGD^*_{Ideal} ($S_{A/B,Ideal}$ vs. $S_{A/B,Theor,BlGr}$)	BD^*_{Ideal} ($S_{A/B,Ideal}$ vs. $S_{A/B,Theor,Gr}$)	
	SD_{Ideal}	$GD_{Ideal} = \frac{GD^*_{Ideal}}{GD^*_{HP}}$	$BGBD_{Ideal} = \frac{BGBD^*_{Ideal}}{BGBD^*_{HP}}$	$BGD_{Ideal} = \frac{BGD^*_{Ideal}}{BGD^*_{HP}}$	$BD_{Ideal} = \frac{BD^*_{Ideal}}{BD^*_{HP}}$	
Real sample	SD^*	GD^* ($S_{A/B}$ vs. $S_{A/B,Theor,Gr}$)	$BGBD^*$ ($S_{A/B}$ vs. $S_{A/B,Theor,BlGrBl}$)	BGD^* ($S_{A/B}$ vs. $S_{A/B,Theor,BlGr}$)	BD^* ($S_{A/B}$ vs. $S_{A/B,Theor,Gr}$)	Target DP
	SD	$GD = \frac{GD^*}{GD^*_{HP}}$	$BGBD = \frac{BGBD^*}{BGBD^*_{HP}}$	$BGD = \frac{BGD^*}{BGD^*_{HP}}$	$BD = \frac{BD^*}{BD^*_{HP}}$	

S2 MODEL DETAILS

The simulations were conducted on a Dell personal computer running Windows 10 (Enterprise, 64-bit), featuring an Intel Core i7-8650U processor (1.90 GHz, 4 cores, BS: 2112 MHz) and 32 GB of RAM. For the Monte Carlo (MC) simulations, which were conducted to generate ideal samples based on pre-determined monomer inclusion probabilities[1, 3, 5] (see **Fig. 2** in main text), the initial number of monomer molecules was set to $1\cdot10^6$. These simulations were conducted over approximately 16 seconds to ensure high reliability. For CMMC simulations, which follow a reaction scheme, the number of monomers was

increased to 2.5·10^7. These simulations were conducted on an hour-scale for each reaction condition, ensuring numerical convergence consistent with previous modeling studies.[6, 7]

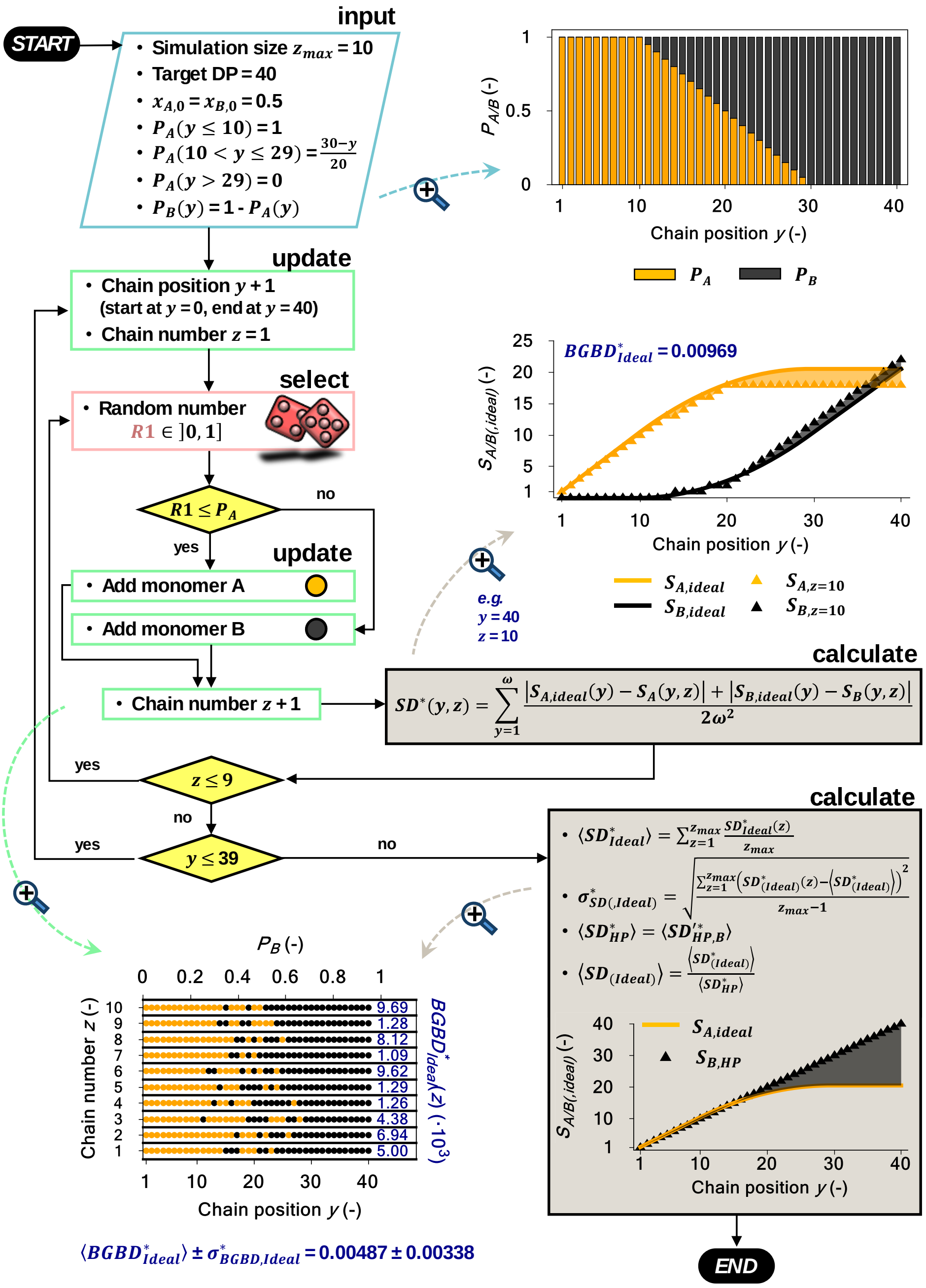

Figure S3. Workflow illustration for generating an ideal Block–Gradient–Block copolymer sample with symmetric gradient and equal-length segments as defined by Equation S2-S4. The simulation begins with defined parameters (top left), including a target DP, symmetric composition ($x_{A,0}$=$x_{B,0}$=0.5), and a monomer inclusion probability profile P_B (top right). At each chain position y, a random number $R1$ determines monomer inclusion based on $P_B(y)$, resulting in individual polymer chains. The cumulative comonomer incorporations $S_A(y,z)$ and $S_B(y,z)$ are then compared to the theoretical profiles $S_{A,ideal}(y)$ and $S_{B,ideal}(y)$ to compute the structural deviation $BGBD^*_{Ideal}(z)$ for each chain z (see also Eq. 1 in main text). Final values for the average deviation $\langle BGBD^*_{Ideal}\rangle$ and its standard deviation $\sigma^*_{BGB,Ideal}$ (see also Eq. 2 and Eq. 3 in main text, respectively) are calculated (bottom right) and the simulated ideal sample is also displayed (bottom left).

S2.1 CATIONIC RING OPENING POLYMERIZATION (CROP)

In **Scheme S1**, the cationic ring opening polymerization (CROP) of poly-2-alkyl/aryl-2oxazolines (PAOx) proceeds through a chain growth process that involves initiation, propagation, and termination steps. The nitrogen atom in the cyclic imino ether group of the 2-oxazoline monomer (M) typically participates in an exothermic, nucleophilic initiation reaction. During this, it attacks the electrophilic initiator (I), causing the initiator to lose its leaving group (X^-), which then acts as a counter ion. Propagation (the growth of polymer P_i with a chain length of i) proceeds through successive nucleophilic attack by incoming monomers on the partially positively charged C5 position of the cationic 2-oxazolinium species. The polymerization can be terminated by the addition of nucleophilic agents.

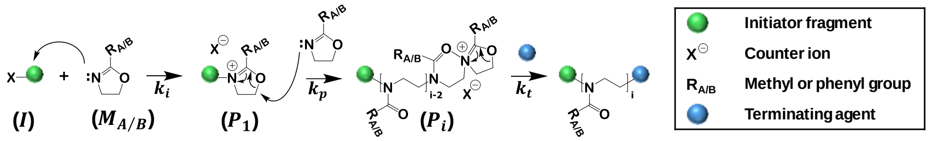

Scheme S1. Main reactions in CROP of two oxazoline monomers (M_A and M_B), leading to targeted linear product.

To minimize the likelihood for a broad molar mass distribution (MMD) and avoid a high dispersity, it is crucial for the initiation of the CROP process to be rapid, similar to reversible-deactivation radical polymerization (RDRP). Past studies have shown that it is ideal for all initiators to begin the chain growth process within 5% of monomer conversion[8] For this reason, methyl tosylate (MeOTs) has been found to be an effective initiator for the process.[9]

S3 EXTRA SIMULATION RESULTS

S3.1 CHARACTERIZING COPOLYMER STRUCTURES THROUGH GD DISTRIBUTION SHAPE

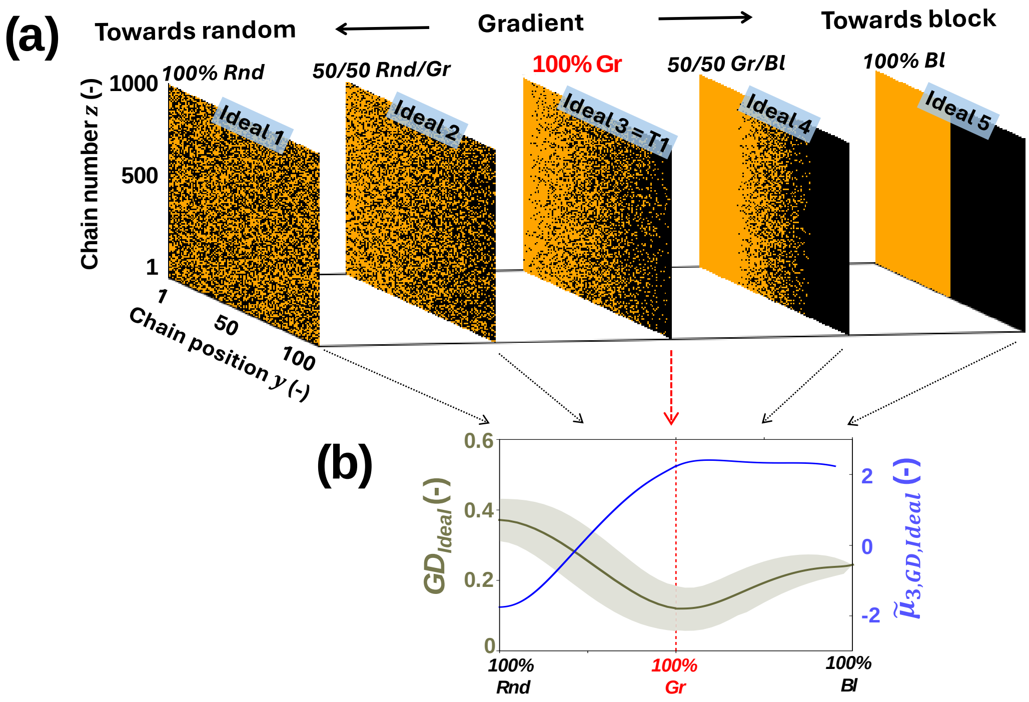

Figure S4. (a) Transforming a targeted gradient structure (Ideal 3=T1; f_{Gr}=1) into random structures (Ideal 1: f_{Rand}=1; and Ideal 2: f_{Gr}=0.5, f_{Rand}=0.5) or ideal block structures (Ideal 4: f_{Gr}=0.5, f_{Bl}=0.5; and Ideal 5: f_{Bl}=1) to highlight the relevance evaluating the GD distribution shape. (b) Non-gradient structures can have similar $\langle GD \rangle \pm \sigma_{GD,Ideal}$ values (brown line) but can still be distinguished by looking at the skewness of these distributions (blue line). $f_{Gr,Bl,Rand}$ is the fraction that is gradient, block, random. It should be noted that in ideal samples where compositional distributions arise solely from the input monomer inclusion probabilities $P_{A/B}$, block copolymers for which these probabilities take discrete (*i.e.* integer) rather than continuous (*i.e.* floating-point) values, exhibit a standard deviation $\sigma_{GD,Ideal}$ of zero. In contrast, σ_{GD} of blocky real copolymers can be high.

S3.2 REFERENCE SIMULATIONS

Table S2. Reactions and ranges for rate coefficients for CROP of 2-oxazoline A (M_A) and 2-oxazoline B (M_B) initiated by MeOTs (I). 100% case: k_{pAA}=0.2 L mol^{-1}s^{-1}.

Reaction	Equation	k-range (k_{pAA}%)	Ref.
Chain initiation	$I + M_A \xrightarrow{k_{i,A}} P_{1,A} + X^-$	$5 \cdot 10^2$	4, 10-12
	$I + M_B \xrightarrow{k_{i,B}} P_{2,A} + X^-$	$5 \cdot 10^2$	4, 10-12

Propagation	$P_{i,A} + M_A \xrightarrow{k_{pAA}} P_{i+1,A}$	$2 \cdot 10^{-1}$	4, 10-12
	$P_{i,B} + M_B \xrightarrow{k_{pBB}} P_{i+1,B}$	$2 \cdot 10^{-1}$	4, 10-12
	$P_{i,A} + M_B \xrightarrow{k_{pAB}} P_{i+1,B}$	$6.67 \cdot 10^{2\,a}$, $2 \cdot 10^{-3\,b}$, $2 \cdot 10^{-1\,c}$, 20^{d}	4, 10-12
	$P_{i,B} + M_A \xrightarrow{k_{pBA}} P_{i+1,A}$	2^{a}, 2^{b}, $2 \cdot 10^{-1\,c}$, 1^{d}	4, 10-12

^{a}P1. ^{b}P2. ^{c} P3. ^{d}P4.

S3.3 EFFECT OF REACTIVITY RATIOS (NO SIDE REACTIONS)

Table S2. Reactions and ranges for rate coefficients for CROP of 2-oxazoline A (M_A) and 2-oxazoline B (M_B) initiated by MeOTs (I). 100% case: k_{pAA}=0.2 L mol^{-1}s^{-1}.

Reaction	Equation	k-range (k_{pAA}%)	Ref.
Chain initiation	$I + M_A \xrightarrow{k_{i,A}} P_{1,A} + X^-$	$5 \cdot 10^2$	4, 10-12
	$I + M_B \xrightarrow{k_{i,B}} P_{2,A} + X^-$	$5 \cdot 10^2$	4, 10-12
Propagation	$P_{i,A} + M_A \xrightarrow{k_{pAA}} P_{i+1,A}$	10^2	4, 10-12
	$P_{i,B} + M_B \xrightarrow{k_{pBB}} P_{i+1,B}$	$1 - 10^2$	4, 10-12
	$P_{i,A} + M_B \xrightarrow{k_{pAB}} P_{i+1,B}$	$1 - 10^4$	4, 10-12
	$P_{i,B} + M_A \xrightarrow{k_{pBA}} P_{i+1,A}$	$1 - 10^4$	4, 10-12

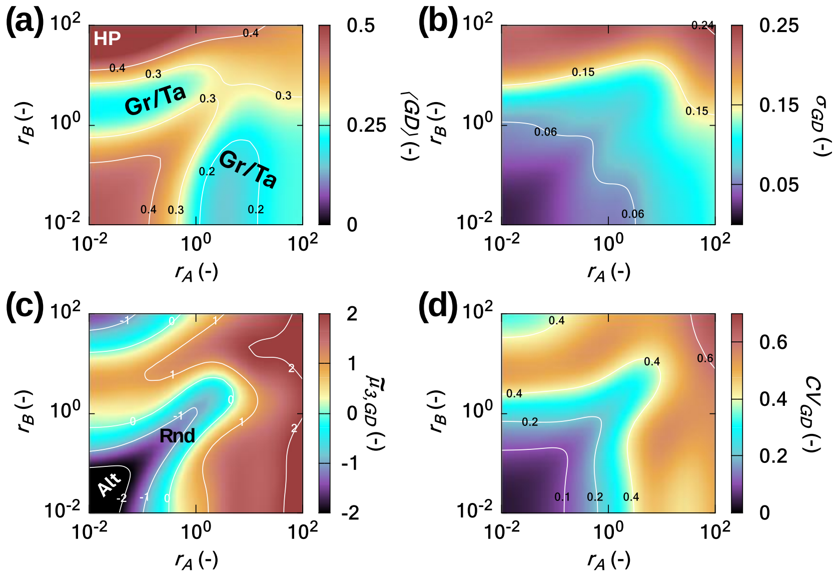

Figure S4. Upgrade of Figure 5 in the main text for k_{pAA}=0.1k_{pBB}.

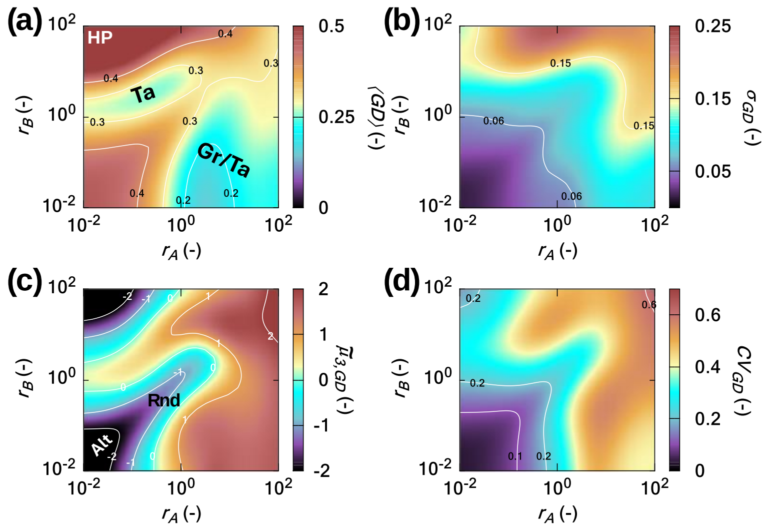

Figure S5. Upgrade of Figure 5 in the main text for k_{pAA}=0.01k_{pBB}.

S3.4 EFFECT OF SIDE REACTIONS AND POLYMERIZATION TEMPERATURE

S3.4.1 Cationic ring opening polymerization (with side reactions)

The CROP of 2-oxazolines can be affected by side reactions, particularly at elevated temperatures used to accelerate the reaction.[13] The most significant side reaction is *β*-elimination, which involves an imine–enamine rearrangement (**Scheme S2b**). In this process, chain transfer to monomer occurs *via* the 2-oxazolinium propagating species.

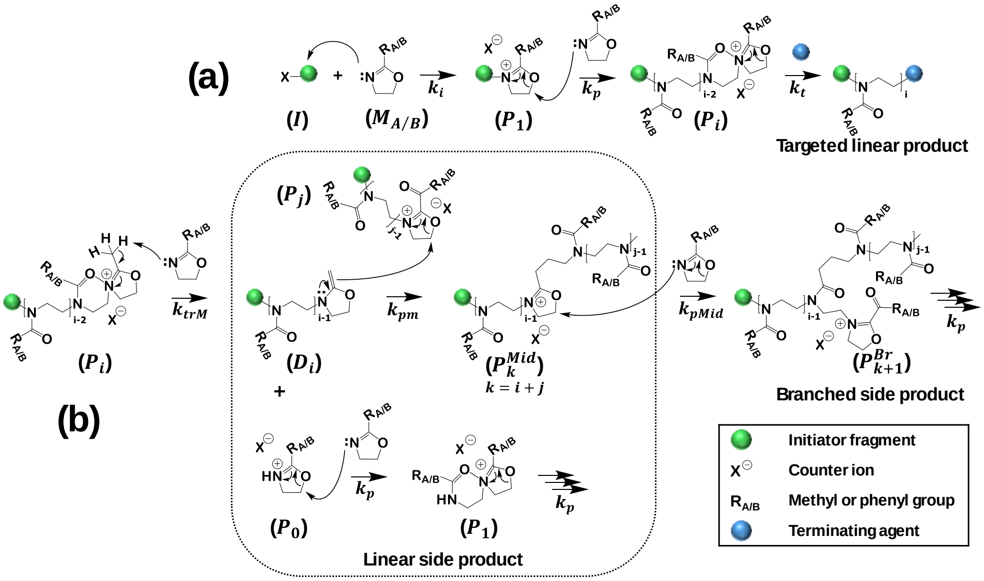

Scheme S2. The CROP of 2-methyl-2-oxazoline (MeOx; M_A) and 2-phenyl-2-oxazoline (PhOx; M_B) yields (a) the targeted linear product and (b) side product, which may be either linear or branched. Chain transfer to monomer (*via* β-elimination) can occur only when MeOx is the terminal unit.

This chain transfer event produces two species: a small, proton-initiated 2-oxazolinium cation (P_0) capable of initiating a new polymer chain, and a larger macromolecular species (D_i). The D_i species, though no longer cationic, features an enamine ether chain end, which allows it to undergo further polymerization through a coupling reaction with an active polymer chain such as P_j.

This macropropagation yields a new linear polymer with a cationic site embedded in the middle of the chain (P_k^{Mid}, with $k = i + j$). As propagation continues, a branched polymer species (P_{k+1}^{Br}) is formed. It is generally assumed that the rate constant for propagation (k_p) of this branched species is the same as that of the linear chains, a typical simplification in models involving branching during polymerization.[10]

Table S3. Reactions and Arrhenius parameters for CROP of MeOx (M_A) and PhOx (M_B) initiated by MeOTs (I), *cf.* Scheme S2.

Reaction	Equation	A (L·mol⁻¹·s⁻¹)	E_A (kJ·mol⁻¹)	Ref.
Chain initiation	$I + M_A \xrightarrow{k_{i,A}} P_{1,A} + X^-$	6.67×10^7	75.4	14
	$I + M_B \xrightarrow{k_{i,B}} P_{1,B} + X^-$	1.49×10^8	84.4	14
Propagation[a]	$P_{i,A} + M_A \xrightarrow{k_{pAA}} P_{i+1,A}$	5.00×10^8	75.4	15
	$P_{i,A}^{Br} + M_A \xrightarrow{k_{pAA}} P_{i+1,A}^{Br}$	5.00×10^8	75.4	15
	$P_{i,B} + M_B \xrightarrow{k_{pBB}} P_{i+1,B}$	1.49×10^9	84.4	15
	$P_{i,B}^{Br} + M_B \xrightarrow{k_{pBB}} P_{i+1,B}^{Br}$	1.49×10^9	84.4	15
	$P_{i,A} + M_B \xrightarrow{k_{pAB}} P_{i+1,B}$	9.55×10^7	80.0	14
	$P_{i,A}^{Br} + M_B \xrightarrow{k_{pAB}} P_{i+1,B}^{Br}$	9.55×10^7	80.0	14
	$P_{i,B} + M_A \xrightarrow{k_{pBA}} P_{i+1,A}$	1.23×10^7	80.0	14
	$P_{i,B}^{Br} + M_A \xrightarrow{k_{pBA}} P_{i+1,A}^{Br}$	1.23×10^7	80.0	14
Chain transfer to monomer (*β*-elimination)	$P_{i,\mathrm{A}} + M_A \xrightarrow{k_{trMAA}} D_{i,\mathrm{A}} + P_{0,\mathrm{A}}$	1.86×10^7 [4]	85.4 [14]	4, 14
	$P_{i,A}^{Br} + M_A \xrightarrow{k_{trMAA}} D_{i,A}^{Br} + P_{0,\mathrm{A}}$	1.86×10^7 [4]	85.4 [14]	4, 14
	$P_{i,\mathrm{A}} + M_\mathrm{B} \xrightarrow{k_{trMAB}} D_{i,\mathrm{A}} + P_{0,\mathrm{B}}$	3.56×10^7 [4]	90.0 [14]	4, 14
	$P_{i,A}^{Br} + M_\mathrm{B} \xrightarrow{k_{trMAB}} D_{i,A}^{Br} + P_{0,\mathrm{B}}$	3.56×10^7 [4]	90.0 [14]	4, 14
Macropropagation[b]	$P_{i,\mathrm{A}} + D_{j,\mathrm{A}} \xrightarrow{k_{pmAA}} P_{i+j,\mathrm{A}}^{Mid}$	5.00×10^7	75.4	4
	$P_{i,\mathrm{B}} + D_{j,\mathrm{A}} \xrightarrow{k_{pmBA}} P_{i+j,\mathrm{A}}^{Mid}$	1.23×10^4	80.0	4
Mid-chain cation propagation	$P_{i+j,\mathrm{A}}^{Mid} + M_\mathrm{A} \xrightarrow{k_{pMidAA}} P_{i+j+1,\mathrm{A}}^{Br}$	5.00×10^8	75.4	12
	$P_{i+j,\mathrm{A}}^{Mid} + M_\mathrm{B} \xrightarrow{k_{pMidAB}} P_{i+j+1,\mathrm{B}}^{Br}$	9.55×10^7	80.0	14

[a] Also for i=0. [b] As the contribution of branched chains (P^{Br} and D^{Br}), particularly macromonomers (D^{Br}), is low macropropagation is assumed to occur only *via* linear species, consistent with the study on the CROP of 2-ethyl-oxazoline by Arraez *et al.*[10]

S3.4.2 Targeted structures

For visualization purposes, 1000 theoretical copolymer chains were considered, each with a degree of polymerization (DP) of 150.[4] Two asymmetric composition profiles were targeted, denoted as T1 and T2, as illustrated in **Fig. S6**. T1 corresponds to an ideal linear gradient copolymer with an overall MeOx:PhOx composition of 70:30. For a chain length of 150, this translates to 105 MeOx and 45 PhOx units. T2 represents a block-gradient copolymer architecture. The first segment is a block consisting of 40% MeOx (or more generally, A-type units), corresponding to 60 monomer units. This is followed by a gradient segment comprising the remaining 60% of the chain (90 monomer units), in which a symmetric 50:50 composition of MeOx and PhOx (A and B) is targeted. This results in an additional 45 MeOx units within the gradient region, maintaining the overall 70:30 MeOx:PhOx ratio.

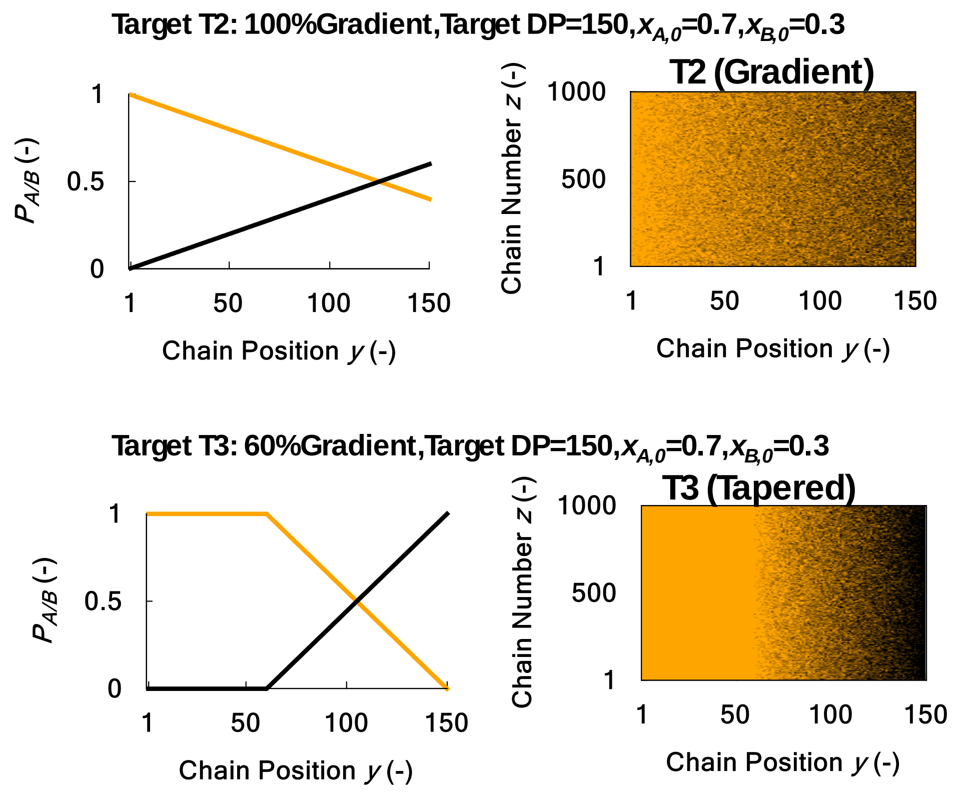

Figure S6. Monomer inclusion probabilities (left) and corresponding ideal polymer samples (right) for gradient (P5, top) and tapered (P6, bottom) structures. Monomer ratio A:B is 105:45, target degree of polymerization (DP) is 150. Monomer A is orange, monomer B is black.

S3.4.3 SD evaluation standard

The SD evaluation standard was developed to provide a quantitative and systematic measure of sequence structure quality. For the original symmetric compositions,[1] the excellent/good threshold ($\langle SD_{Good/Poor}\rangle$=0.06) was analytically determined as the average of an ideal symmetric linear gradient copolymer. The good/poor threshold ($\langle SD_{Good/Poor}\rangle$=0.3), by contrast, was chosen arbitrarily as a practical reference **but supported by inspection of plots of explicit monomer sequences**. For asymmetric compositions (70/30),[4] these thresholds were adapted accordingly, recognizing that the distribution statistics differ from the symmetric case.

Only in a subsequent publication,[5] a framework was developed to interpret all SD thresholds analytically, allowing the numbers to be connected rigorously to chain statistics rather than chosen heuristically. Overall, the standard provides a reproducible, interpretable, and quantitative criterion for classifying chain quality across different compositions. Here, the 98th percentile of the SD distribution was also used to define the upper limit of good chains and the lower limit of poor chains, providing an analytical solution to statistical reference $\langle SD_{Good/Poor}\rangle$.

The threshold SD parameters $\langle SD_{Good/Poor}\rangle$ = and $\langle SD_{Good/Poor}\rangle$ are given by **Equation** $S23$ and **Equation** $S24$ respectively:

$\langle SD^{(*)}_{Exc/Good}\rangle = \langle SD^{(*)}_{Ideal}\rangle$	$(S23)$
$\langle SD^{(*)}_{Good/Poor}\rangle = SD^{(*)}_{Ideal,98\%}$	$(S24)$

Figure S7 shows the SD distributions of T2 and T3 and illustrates how these data can be translated into statistical descriptors of chain quality.

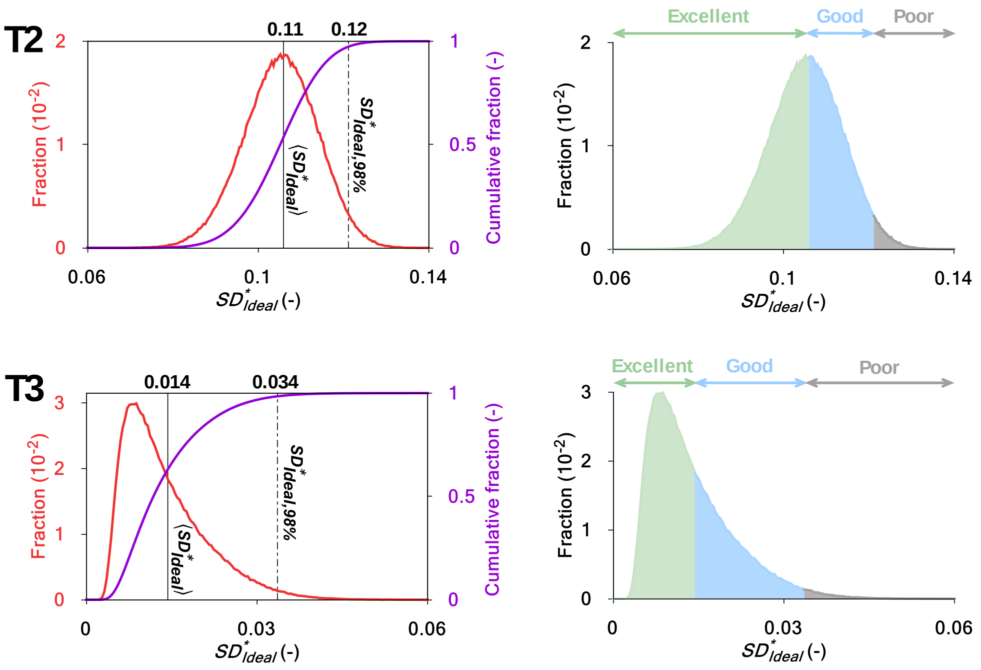

Figure S7. Evaluation of T2 (top) and T3 (bottom) on their SD^*_{Ideal} distributions. Left column: number fraction (red) and cumulative fraction (purple) SD^*_{Ideal} densities. The average values $\langle SD^*_{Ideal}\rangle$ (solid black lines) are 0.11 for T2 and 0.014 for T3. These average values correspond to threshold SD values in the right column, defining the limits for chain quality: $\langle SD^*_{Exc/Good}\rangle$ marks the upper limit for "excellent" chains (green) and the lower limit for "good" chains (blue). The 98th percentile values, $SD^*_{Ideal,98\%}$ (dash-dotted lines), are 0.12 for T2 and 0.034 for T3. These percentile values correspond to the thresholds $\langle SD^*_{Good/Poor}\rangle$in the right column, marking the upper limit for "good" chains (blue) and the lower limit for "poor" chains (gray). Note that changes as small as 0.01 in $SD^*_{Ideal}$are significant.

Figure S8 extends this analysis to examine how gradient percentage, initial B fraction, and target DP influence these quality descriptors. **Figure S9** presents a simplified version of Figure S8, focusing exclusively on the quality descriptors for clarity, but from a more practical view where the normalized SD values are used.

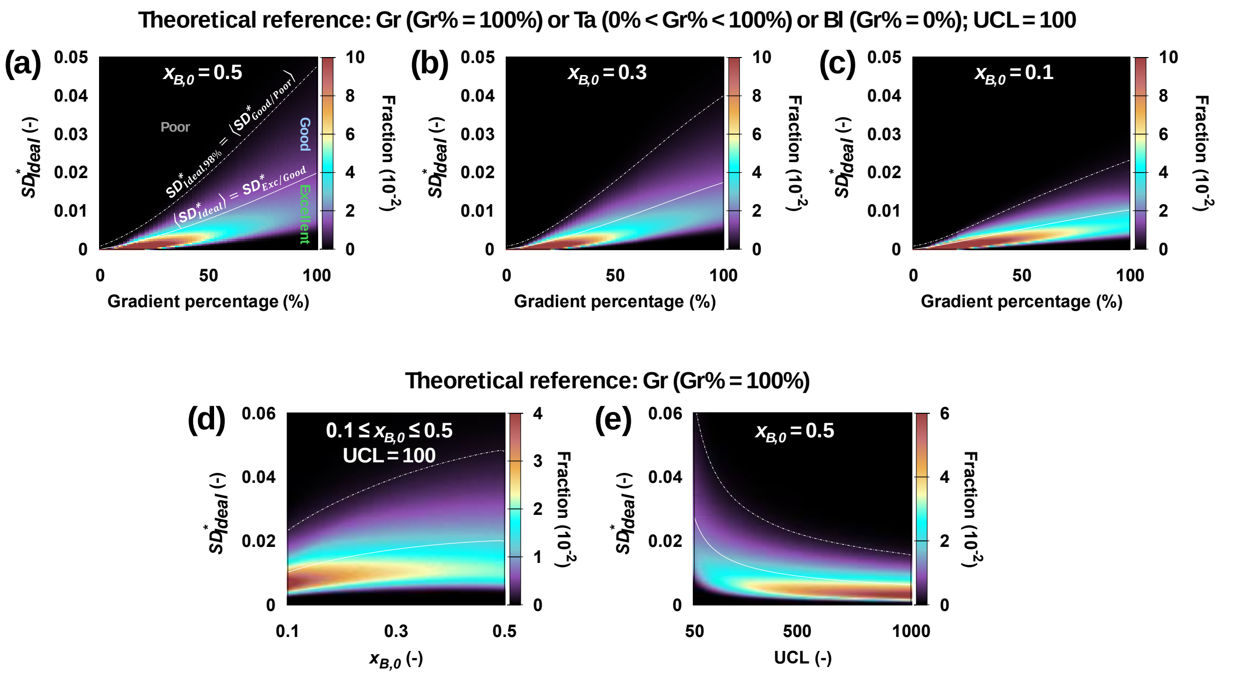

Figure S8. Upgrade of Figure 3 in the main text, showing the threshold parameters $\langle SD^*_{Exc/Good}\rangle$(solid line) and $\langle SD^*_{Good/Poor}\rangle$ (dash-dotted line).

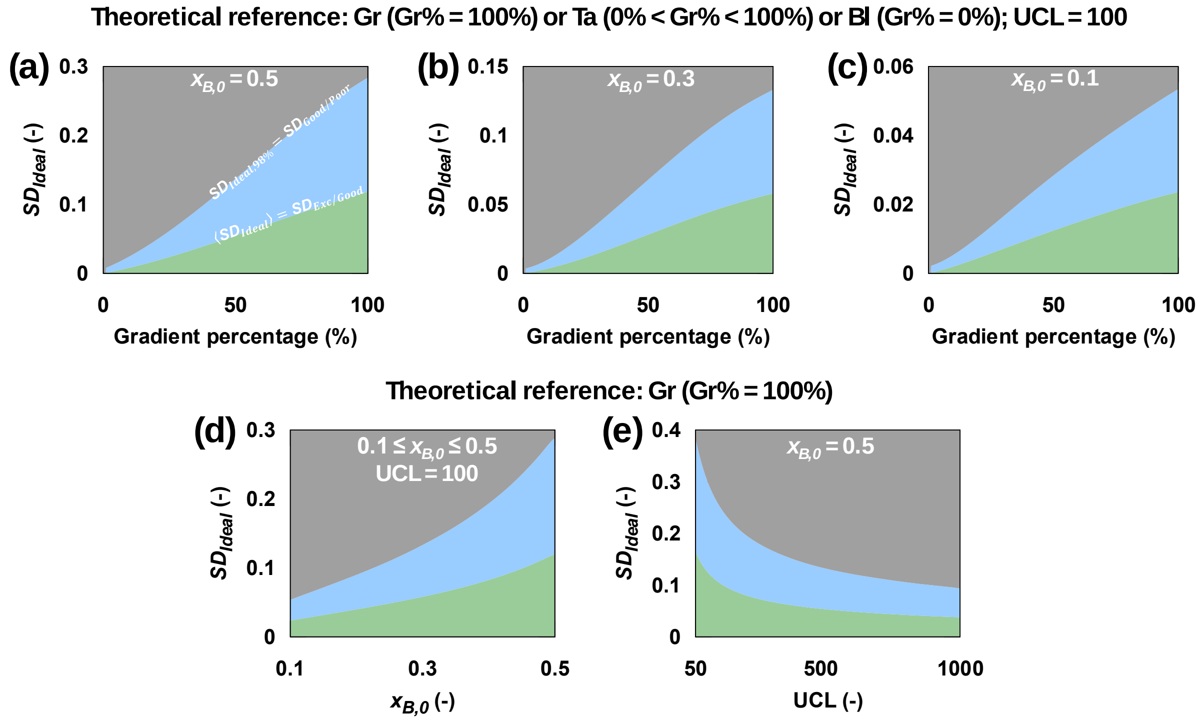

Figure S9. Updated version of Figure S8 showing the normalized SD_{Ideal} (see Step 3 in Section S1.3 of the Supporting Information). Chains are categorized by quality: "excellent" (green: $0 \leq SD \leq \langle SD_{Exc/Good}\rangle$), "good" (blue: $\langle SD_{Exc/Good}\rangle < SD \leq \langle SD_{Good/Poor}\rangle$), and "poor" (gray: $SD > \langle SD_{Good/Poor}\rangle$).

S3.4.4 Branched chain algortihm

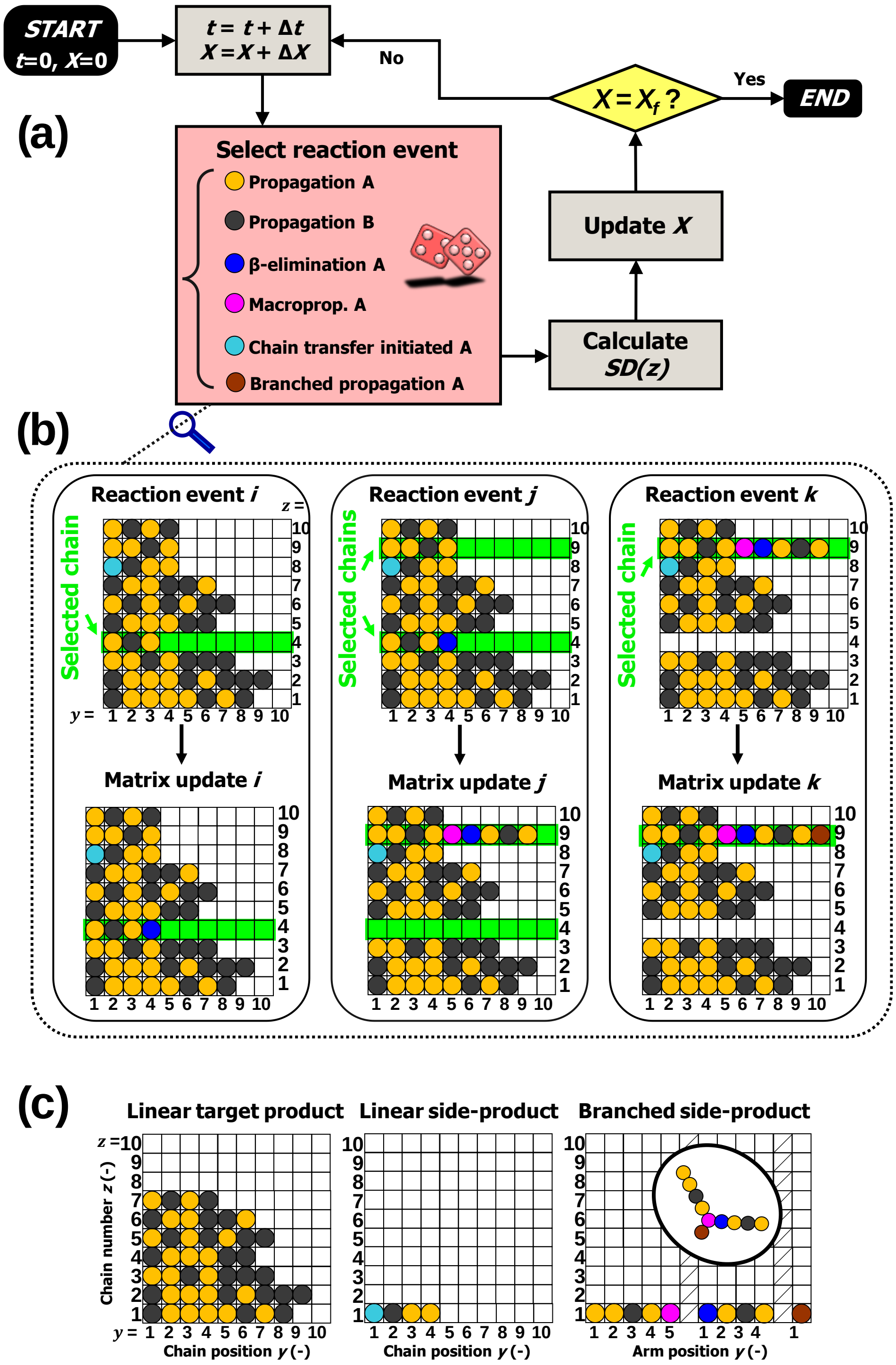

Figure S10. (a) Coupled-matrix Monte Carlo (CMMC) flowsheet focusing only on the reaction events building up the product matrices. (b) Close-up of reaction events and their matrix representation, showcasing three hypothetical events: *i* (β-elimination of chain 4, randomly selected), *j* (micropropagation, randomly selecting chains 4 and 9, with chain 4 added to chain 9, resulting in an empty 4th row in the matrix), and *k* (branched

propagation of monomer A, involving chain 9, the only eligible chain). (c) Post-processing of CMMC matrices to derive linear target product, linear side product, and branched side product.

S4 REFERENCES

1. Van Steenberge Paul, H.; D’hooge Dagmar, R.; Yu, W.; Mingjiang, Z.; Marie-Françoise, R.; Dominik, K.; Krzysztof, M., Linear Gradient Quality of ATRP Copolymers. **2012**.
2. Fierens, S. K.; Van Steenberge, P. H.; Reyniers, M. F.; Marin, G. B.; D'hooge, D. R., How penultimate monomer unit effects and initiator influence ICAR ATRP of n-butyl acrylate and methyl methacrylate. *AIChE Journal* **2017,** *63* (11), 4971-4986.
3. Toloza Porras, C.; D'hooge, D. R.; Van Steenberge, P. H.; Reyniers, M. F.; Marin, G. B., A theoretical exploration of the potential of ICAR ATRP for one-and two-pot synthesis of well-defined diblock copolymers. *Macromolecular Reaction Engineering* **2013,** *7* (7), 311-326.
4. Conka, R.; Marien, Y. W.; Sedlacek, O.; Hoogenboom, R.; Van Steenberge, P. H.; D'hooge, D. R., A unified kinetic Monte Carlo approach to evaluate (a) symmetric block and gradient copolymers with linear and branched chains illustrated for poly (2-oxazoline) s. *Polymer Chemistry* **2022,** *13* (11), 1559-1575.
5. Conka, R.; Marien, Y. W.; Van Steenberge, P. H.; Hoogenboom, R.; D'hooge, D. R., An equation driven quality classification of (a) symmetric gradient, gradient-block, block-gradient-block and block copolymers. *European Polymer Journal* **2023,** *185*, 111769.
6. Fierens, S. K.; D’hooge, D. R.; Van Steenberge, P. H.; Reyniers, M.-F.; Marin, G. B., MAMA-SG1 initiated nitroxide mediated polymerization of styrene: From Arrhenius parameters to model-based design. *Chemical Engineering Journal* **2015,** *278*, 407-420.
7. Payne, K. A.; D’hooge, D. R.; Van Steenberge, P. H.; Reyniers, M.-F.; Cunningham, M. F.; Hutchinson, R. A.; Marin, G. B., ARGET ATRP of butyl methacrylate: utilizing kinetic modeling to understand experimental trends. *Macromolecules* **2013,** *46* (10), 3828-3840.
8. D'hooge, D. R.; Konkolewicz, D.; Reyniers, M. F.; Marin, G. B.; Matyjaszewski, K., Kinetic modeling of ICAR ATRP. *Macromolecular theory and simulations* **2012,** *21* (1), 52-69.
9. Hoogenboom, R.; Fijten, M. W.; Schubert, U. S., Parallel kinetic investigation of 2-oxazoline polymerizations with different initiators as basis for designed copolymer synthesis. *Journal of Polymer Science Part A: Polymer Chemistry* **2004,** *42* (8), 1830-1840.
10. Arraez, F. J.; Xu, X.; Van Steenberge, P. H. M.; Jerca, V.-V.; Hoogenboom, R.; D’hooge, D. R., Macropropagation Rate Coefficients and Branching Levels in Cationic Ring-Opening Polymerization of 2-Ethyl-2-oxazoline through Prediction of Size Exclusion Chromatography Data. *Macromolecules* **2019,** *52* (11), 4067-4078.
11. Bouten, P. J. M.; Hertsen, D.; Vergaelen, M.; Monnery, B. D.; Catak, S.; van Hest, J. C. M.; Van Speybroeck, V.; Hoogenboom, R., Synthesis of poly(2-oxazoline)s with side chain methyl ester functionalities: Detailed understanding of living copolymerization behavior of methyl ester containing monomers with 2-alkyl-2-oxazolines. *Journal of Polymer Science Part A: Polymer Chemistry* **2015,** *53* (22), 2649-2661.
12. Wiesbrock, F.; Hoogenboom, R.; Leenen, M.; van Nispen, S. F.; van der Loop, M.; Abeln, C. H.; van den Berg, A. M.; Schubert, U. S., Microwave-assisted synthesis of a 42-membered library of diblock copoly (2-oxazoline) s and chain-extended homo poly (2-oxazoline) s and their thermal characterization. *Macromolecules* **2005,** *38* (19), 7957-7966.

13. Monnery, B. D.; Jerca, V. V.; Sedlacek, O.; Verbraeken, B.; Cavill, R.; Hoogenboom, R., Defined high molar mass poly (2-oxazoline) s. *Angewandte Chemie International Edition* **2018,** *57* (47), 15400-15404.
14. Van Steenberge, P. H. M.; Verbraeken, B.; Reyniers, M.-F.; Hoogenboom, R.; D'hooge, D. R., Model-Based Visualization and Understanding of Monomer Sequence Formation in Gradient Copoly(2-oxazoline)s On the basis of 2-Methyl-2-oxazoline and 2-Phenyl-2-oxazoline. *Macromolecules* **2015,** *48* (21), 7765-7773.
15. Wiesbrock, F.; Hoogenboom, R.; Leenen, M.; van Nispen, S. F. G. M.; van der Loop, M.; Abeln, C. H.; van den Berg, A. M. J.; Schubert, U. S., Microwave-Assisted Synthesis of a 42-Membered Library of Diblock Copoly(2-oxazoline)s and Chain-Extended Homo Poly(2-oxazoline)s and Their Thermal Characterization. *Macromolecules* **2005,** *38* (19), 7957-7966.
